# Epigenetic biotypes of post-traumatic stress disorder in war-zone exposed veteran and active duty males

**DOI:** 10.1038/s41380-020-00966-2

**Published:** 2020-12-18

**Authors:** Ruoting Yang, Aarti Gautam, Derese Getnet, Bernie J. Daigle, Stacy Miller, Burook Misganaw, Kelsey R. Dean, Raina Kumar, Seid Muhie, Kai Wang, Inyoul Lee, Duna Abu-Amara, Janine D. Flory, The PTSD Systems Biology Consortium, Allison Hoke, Nabarun Chakraborty, Linda Petzold, Gwyneth Wu, Guia Guffanti, Taek-Kyun Kim, Min Young Lee, Linda Bierer, Leroy Hood, Owen M. Wolkowitz, Synthia H. Mellon, Francis J. Doyle, Rachel Yehuda, Charles R. Marmar, Kerry J. Ressler, Rasha Hammamieh, Marti Jett

**Affiliations:** 1 Medical Readiness Systems Biology, Walter Reed Army Institute for Research, Silver Spring, MD USA; 2 Advanced Biomedical Computation Sciences, Frederick National Laboratory for Cancer Research, Frederick, MD USA; 3 Departments of Biological Sciences and Computer Science, The University of Memphis, Memphis, TN USA; 4 Harvard John A. Paulson School of Engineering and Applied Sciences, Harvard University, Cambridge, MA USA; 5 Department of Systems Biology, Harvard University, Cambridge, MA USA; 6 Institute for Systems Biology, Seattle, WA USA; 7 Department of Psychiatry, New York Langone Medical School, New York, NY USA; 8 Department of Psychiatry, James J. Peters VA Medical Center, Bronx, NY USA; 9 Department of Psychiatry, Icahn School of Medicine at Mount Sinai, New York, NY USA; 10 Department of Psychiatry, University of California, San Francisco, CA USA; 11 Department of Obstetrics, Gynecology & Reproductive Sciences, University of California, San Francisco, CA USA; 12 McLean Hospital, Belmont, MA USA; 13 Harvard Medical School, Boston, MA USA; 14 Department of Computer Science, University of California, Santa Barbara, CA USA

**Keywords:** Psychology, Genetics

## Abstract

Post-traumatic stress disorder (PTSD) is a heterogeneous condition evidenced by the absence of objective physiological measurements applicable to all who meet the criteria for the disorder as well as divergent responses to treatments. This study capitalized on biological diversity observed within the PTSD group observed following epigenome-wide analysis of a well-characterized Discovery cohort (*N* = 166) consisting of 83 male combat exposed veterans with PTSD, and 83 combat veterans without PTSD in order to identify patterns that might distinguish subtypes. Computational analysis of DNA methylation (DNAm) profiles identified two PTSD biotypes within the PTSD+ group, G1 and G2, associated with 34 clinical features that are associated with PTSD and PTSD comorbidities. The G2 biotype was associated with an increased PTSD risk and had higher polygenic risk scores and a greater methylation compared to the G1 biotype and healthy controls. The findings were validated at a 3-year follow-up (*N* = 59) of the same individuals as well as in two independent, veteran cohorts (*N* = 54 and *N* = 38), and an active duty cohort (*N* = 133). In some cases, for example Dopamine-PKA-CREB and GABA-PKC-CREB signaling pathways, the biotypes were oppositely dysregulated, suggesting that the biotypes were not simply a function of a dimensional relationship with symptom severity, but may represent distinct biological risk profiles underpinning PTSD. The identification of two novel distinct epigenetic biotypes for PTSD may have future utility in understanding biological and clinical heterogeneity in PTSD and potential applications in risk assessment for active duty military personnel under non-clinician-administered settings, and improvement of PTSD diagnostic markers.

## Introduction

Post-traumatic stress disorder (PTSD) is a stress-related syndrome that develops in many following exposure to serious or life-threatening traumatic events [1, 2]. At the current time, PTSD diagnoses are based on self-reports of the frequency and severity of both psychological and physiological symptoms, which are subject to witting or unwitting over- and under-reporting. As such, it has been a major priority to identify objective biological markers to aid and increase diagnostic and prognostic accuracy [3], and many such efforts have been ongoing in recent years [4].

Although a myriad of biological differences has been noted between groups of trauma survivors with or without PTSD, a bona fide diagnostic test for this disorder that provides high sensitivity and specificity has been elusive. One of the major barriers to identifying PTSD biomarkers is that as more symptoms are added to the diagnosis, the number of different PTSD presentations is also increased, resulting in a remarkably heterogeneous disorder. There have been efforts to symptomatically classify PTSD into distinct pathological post-traumatic phenotypes, resulting in an official dissociative subtype in the DSM-5 [5, 6]. The comorbidity PTSD/MDD phenotype is also believed to be a more severe subtype [2]. The approach used for subtype detection is based on identifying different biological correlates associated with symptoms that seemed to be present in only a subset of individuals with PTSD. For example, Lanius et al. estimated that the PTSD patient population could be divided into 70% with a re-experiencing/hyperarousal subtype and 30% with a dissociative subtype [7], and these subtypes showed distinct activations in the medial prefrontal brain regions using functional magnetic resonance imaging (fMRI) [8, 9]. Moreover, people with the same symptom may have different underlying mechanisms contributing to symptom expression [10]. Recently Drysdale et al. identified ‘biotypes’ from objective biological measurements (fMRI in this case), and the biotypes associated with the clinical/symptomatic correlates [11, 12]. The biotypes stratified the biological variation resulting in different clinical phenotypes and hereby inferred more targeted/personalized objective markers and treatment. Therefore, the current study attempted to identify PTSD biotypes using blood epigenome-wide array data.

DNA methylation (DNAm) is a cellular process of epigenetic regulation employed by cells to control gene expression without altering the genetic sequence. Commercial epigenome-wide arrays (Illumina series) have been proven to be highly reproducible and yielded the identification of DNAm markers that can be accurate and stable enough to estimate white blood cell composition [13], smoking history [14], and age [15–17]. Moreover, there is growing evidence that epigenetic processes play an important role in the etiology of psychological disorders [18] and PTSD [19–22].

There have been many attempts to identify DNAm markers using blood epigenome-wide array [20, 21, 23–28]. While significant group differences have been noted, these studies have failed to demonstrate the presence of any single or group of robust and accurate markers in diagnosing PTSD, that would be applicable to all patients with this condition [29]. The failure to identify a stronger signal may have to do with potential biotypes that contribute in opposite directions, and many proposed markers can be biotype-specific. If so, comparing the whole epigenome between PTSD and controls might wash out potential differences within biotypes, or yield significant differences as a function of specific clinical characteristics of the sample being studied. To further examine this possibility, blood epigenome data from a carefully characterized veteran cohort were subjected to computational analyses to determine whether different biotypes could be discerned, and if so, whether these were associated with different clinical/symptom correlates (Fig. S1). The biotypes were then evaluated in three independent samples, including a 3-year follow-up cohort of the original, for purposes of independent validation. As a further confirmation of their potential clinical and screening utility, the biotypes were examined in an active duty sample before and after deployment. To address issues of the specificity of the marker for PTSD, difference between a comorbid PTSD/MDD (Major Depression Disorder) and MDD alone was investigated using an independent MDD cohort. Finally, it was of interest to explore the association between identified biotypes and PTSD symptoms, polygenic risk scores (PRSs), biological pathways, and biomarkers.

## Result

### Epigenetic profile revealed two PTSD biotypes

**Fig. 1:**
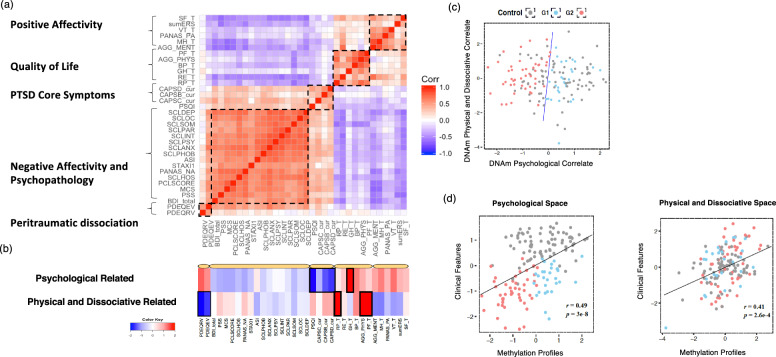
Two epigenetic biotypes G1 and G2 were identified based on 100 clinical-meaningful DNAm genes. **a** Thirty-four well-characterized symptomatic assessments (Table S1) were grouped into five clusters based on Pearson’s correlations values; we assigned clinical-related names to these groups (labels on the left). The values were translated to a color gradient schema in which, red indicates a value of 1 (i.e., positive correlation) and blue indicates −1 (i.e., negative correlation). **b** Heatmap illustrating the *z*-scores of the components of two CCA latent clinical factors. The first factor is focused on PTSD core symptoms, named ‘Psychological’, and the second factor contains physical and dissociative, named ‘Physical and dissociative’. Colors span dark blue to dark red where dark blue denotes a *z*-score of −2, and dark red indicates a *z*-score of 2. Black boxes were added to highlight the components highly ranked based on absolute *z*-scores. **c** The scatter plot shows the DNAm Psychological and Physical score paired with their associated Psychological and Physical clinical features. The highly correlated pairs were identified by PCA + CCA approach based on Discovery cohort (*N* = 157, *r* = 0.49, *p* = 3e−8, and *r* = 0.41, *p* = 2.6e−4, respectively). The gray dots are controls, and PTSD individuals are presented in colored dots. **d** LDA classifier was trained on DNAm ‘Psychological’ and ‘Physical and dissociative’ to assign any PTSD individuals to G1 or G2 biotypes. The gray dots are controls, the blue and red dots are PTSD G1 and G2 biotype.

The epigenome-wide analysis was initiated in a combat-exposed male veteran Discovery cohort (*N* = 166) consisting of 83 PTSD+ and 83 PTSD− participants with 34 clinically meaningful feature assessments (Table S1), which were divided into five functional clusters (Fig. 1a). A hundred gene regions associated with these clinical features were identified (Table S2), and proceeded to the Principal Component Analysis (PCA) + Canonical Correlation Analysis (CCA) approach (see Method) to achieve two gene-clinical correlates (*r* = 0.49, *p* value = 3.0e−8, and *r* = 0.41, *p* value = 2.6e−4) (Fig. 1c). The clinical component of the first correlate named the ‘Psychological’, was comprised of PTSD core symptoms, the other, named the ‘Physical and Dissociative’, was comprised of quality of life and peri-traumatic dissociation (Fig. 1b). This linkage of physical and dissociative characteristics is supported by Van der Hart et al.’s proposition of positive (trauma-related physical pain) and negative (functional losses such as amnesia and paralysis) *somatoform* manifestations of dissociation [30]. Based on the correlates, the clinical features of each participant can be characterized in two-dimensional DNAm ‘Psychological’ and ‘Physical and Dissociative’ space. A Linear Discriminant Analysis (LDA) classifier was used to separate PTSD and controls (Fig. 1c). A group of PTSD individuals (in blue) were highly overlapped with controls (in gray) with a negative subtype score, named G1 epigenetic biotype; while the other PTSD individuals (in red) were distinct with controls with a positive subtype score, named G2. Importantly, new cases can be assigned as G1 or G2 based on their DNAm biotype scores (see Method). As shown in Fig. 1d, G1, G2, and the control were three distinct clinical phenotypes in the psychological space but were mixed in the physical-dissociative space. Overall, PTSD cases reported more extreme physical health than the control, while more G2 biotypes reported extreme health (clinical PD score >1 or < −1) than G1 (25 vs. 10). These observations imply that the biotypes reflect not only severity difference of CAPS, but also a comprehensive combination of symptom and functional ratings.

### Biotypes are preserved in Replication cohorts

**Fig. 2:**
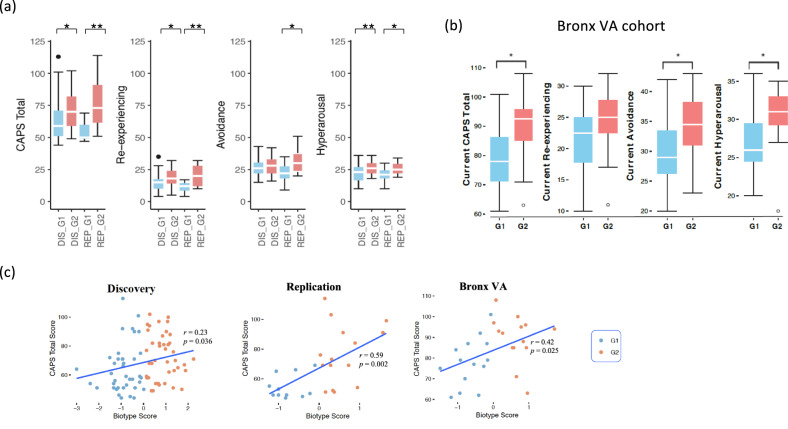
Individuals of the G2 biotype have significantly higher PTSD severity than G1. **a** We compared the difference in CAPS scores between the PTSD individuals in our Discovery (DISC) (G1, *N* = 39; G2, *N* = 41) and Replication (REP) (G1, *N* = 11; G2, *N* = 15) cohorts. The statistical significance was defined by a two-tailed *t*-test and double asterisks indicate *p* < 0.005, asterisk indicates *p* < 0.05. **b** PTSD severity comparison in the veteran Bronx VA cohort. Comparison of current and lifetime CAPS scores between the PTSD individuals in veteran Bronx VA cohort (G1, *N* = 14; G2, *N* = 14). **c** Correlation between biotype score and CAPS score for different cohorts.

To further test the biotypes, the biotypes, G1 and G2, were assigned to the PTSD cases of the Replication cohort (*N* = 26). Fig. 2a showed that the G2 subgroup had a consistently higher Clinician-Administered PTSD Scale (CAPS) [31] total score and CAPS subcategory scores as compared to the G1 subgroup both in the Discovery and Replication cohorts. The biotypes were further evaluated using the second Replication cohort, Bronx VA cohort with 28 PTSD veterans who have moderate/severe PTSD symptoms. A comparison of the resulting biotype groups revealed a similar pattern observed in the prior analysis (Fig. 2b). Although the CAPS was significantly associated with biotype scores (see method, Fig. 2c), CAPS difference is not enough to explain the variance of biotypes. Deeper inherent differences are expected between biotypes.

### Biotype’s potential to predict PTSD risk is retained in the 3-year follow-up

**Fig. 3:**
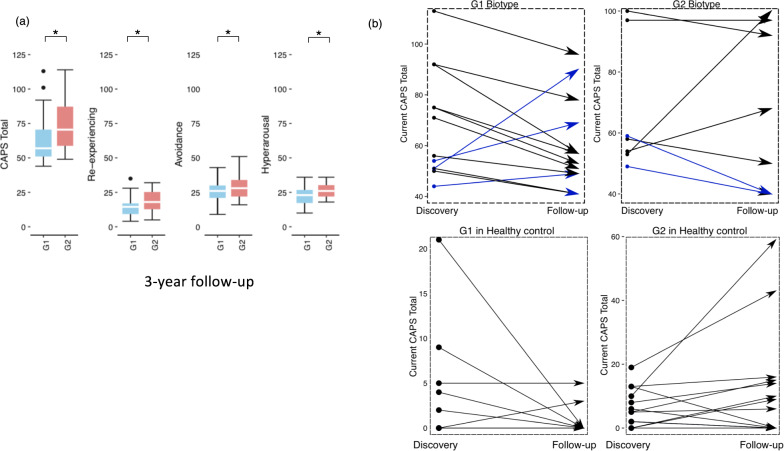
Validation in the 3-year Follow-up cohort. **a** The CAPS scores between G1 and G2 in the Follow-up cohort (G1, *N* = 11; G2, *N* = 12) were compared. **b** Plot illustrates the Current CAPS total scores at Discovery and the 3-year Follow-up time points for the 19 (out of 83) PTSD-positive individuals and 29 PTSD negative individuals (at Discovery) with data for both time point.

The potential of two biotypes associated with PTSD risk was further tested in the Follow-up cohort including 59 individuals originally part of the Discovery who returned for an average 3-year longitudinal sample collection and clinical evaluation. Of those 59 individuals, 23 were identified as PTSD positive or high subthreshold CAPS scores (CAPS ≥ 40) upon recall. Nineteen of 23 individuals were subtyped in the Discovery (12 G1 and 7 G2). To determine if the DNAm biotyping is sensitive to the symptom changes, these 23 individuals were re-biotyped resulting in 13 G1 and 10 G2. Consistent with prior observations, G2 had significantly higher CAPS scores than G1 (Fig. 3a). All three individuals who were G1 biotype at Discovery and elevated their CAPS score upon follow-up in Fig. 3b have switched to G2, while the two original G2 PTSD positives became subthreshold upon recall have switched to G1.

**Fig. 4:**
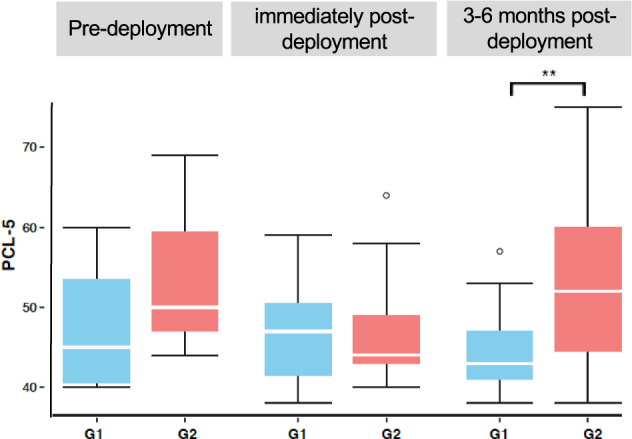
Validation in the active duty Fort Campbell cohort. Comparison of the PCL-5 score of PTSD-positive G1 and G2 individuals from the active duty Fort Campbell cohort pre-deployment (G1, *N* = 7 and G2, *N* = 3), 3-days returning from duty (G1, *N* = 11 and G2, *N* = 10) and 3–6 months post-deployment (G1, *N* =23 and G2, *N* = 22).

To investigate the effect of biotypes on individuals that were classified as PTSD negative (current CAPS scores <20, one patient has 22), biotypes were assigned to the 82 PTSD negative individuals in the Discovery, and no CAPS difference was found between biotypes (G1: *N* = 40, G2: *N* = 42, *p* = 0.879). A closer look at 29 of the 82 PTSD negative individuals that were evaluated at follow-up, the G1 biotype (*N* = 11) showed an overall decrease of CAPS total score, while the G2 biotype (*N* = 18) showed an overall increase of CAPS score with two individuals becoming PTSD positive or subthreshold (Fig. 4). These results raise the possibility that the biotypes may be associated with PTSD susceptibility.

### Biotypes are promising for screening in active duty

To determine whether biotypes could be used as a screening tool to active duty service members, the biotyping was applied to a longitudinal active duty cohort collected and assessed in Fort Campbell, Kentucky. Participants were evaluated using the self-report PTSD Checklist for DSM-5 (PCL-5) with 76 PTSD-positive cases (PCL ≥ 38), including 10 in pre-deployment, 21 in post-deployment, and 45 in 3–6 months post-deployment. The ten participants in pre-deployment already showed a high PCL score, likely due to the previous deployments. Consistent with the findings in the veteran cohorts, the PCL score in G2 biotype was significantly higher than G1in 3–6 months post-deployment. The pre-deployment was similar, although not significantly so, due to the sample size. However, the PCL difference was not seen to occur immediately after deployment (Fig. 4), possibly because post-traumatic stress often onsets after 1 month or later. Thirteen of 21 PTSD-positive subjects in post-deployment retested in 3–6 months. Four individuals belonging to the G1 type reduced their PCL-5 scores under 38 (3 have dropped more than 20).

### Biotypes associate with anxiety/depressive symptoms

Combining the Discovery and Replication cohorts, we further compared the difference in a total of 49 clinical features (Table S3), including the aforementioned 34 clinical features, as well as lifetime CAPS, and early life experience scores, between the two biotypes adjusting for BMI and age.

**Fig. 5:**
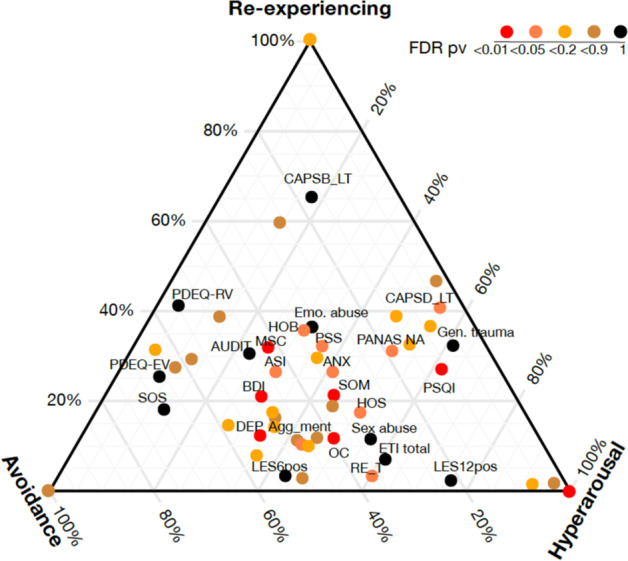
The biotypes differentiate in anxiety and depressive symptoms. The ternary plot shows the relative similarity of each clinical feature to three PTSD core subcategories. The statistical significance of the difference between the two biotypes is colored from red to black (red is for false discovery rate (FDR) < 0.01, black for FDR = 1, with other colors in between as denoted in the figure). The clinical features with significant biotype difference (FDR < 0.05) and no difference (FDR = 1) were labeled.

The ternary plot in Fig. 5 showed the correlation of each feature (dot) to the three current CAPS subcategories by its relative distance to the three vertices, and the significance of biotype difference for each clinical feature was highlighted in color. Compared to G1, biotype G2 exhibited significantly more anxiety symptoms (e.g. CAPS hyperarousal scores (CAPSD_cur), Mississippi Scale for Combat (MSC) [32], Pittsburgh Sleep Quality Index (PSQI) [33], and Symptom Checklist 90 [34] (SCL)-somatization anxiety, hostility, obsession-compulsive), and depressive symptoms (measured by BDI-II depression score [35] and SCL Depression)) (red or coral dots). No statistical biotype difference was found in the level of current re-experience (CAPSB) and avoidance (CAPSC) (brown dots). Moreover, no difference in lifetime re-experiencing/avoidance (CAPSB_LT, CAPSC_LT) or early trauma (Early Trauma Inventory (ETI): General trauma, emotional abuse, and sex abuse [36]) was observed (black dots).

The MDD/PTSD comorbidity phenotype (defined by CAPS ≥ 40 and BDI ≥ 14) is highly enriched in G2 type (MDD/PTSD percentage in G1 vs. G2 = 71.8% vs. 97.6%, BDI of G1 vs. G2: 20.5 vs. 30.9, *p* = 0.0002) than G1.In the medication history, blood pressure medicines, pain killers, antidepressants, and sleep pills were more often used in the G2 type (Table S4), which has more physical comorbidities (bodily pain (BP_T, less score indicates more pain) 45.9 vs. 35.8, *p* = 0.0133), and more comorbid MDD.

To validate that the G1/G2 difference is not driven by depression comorbidity, G1 and G2 were categorized in a civilian cohort (MDD UCSF cohort) characterizing MDD with no PTSD comorbidity (27 MDD males and 22 MDD females) and found no depression severity difference between the Hamilton Depression Rating Scale (HAM-D) of the biotypes (*p* = 0.706 for males and 0.741 for females, Fig. S2).

### Biotypes associate with Polygenic Risk Scores (PRS)

Polygenic analysis has also been used to investigate disease heterogeneity and subtypes [37, 38], thereby we compared PRS between two biotypes. Combining Discovery and Replication cohorts, the PRS is modestly correlated with current CAPS total (*r* = 0.12, *p* = 0.061). The G2 biotype has a modestly higher PRS than the G1 biotype (*N* = 106, G1 vs. G2: mean 120.77 vs. 138.98, *p* = 0.233). However, PRS is significantly higher in G2 when excluding the participants with African ancestry (*N* = 70, mean 88.29 vs. 131.30, *p* = 0.018), since PRS of PGC-PTSD Freeze 2 cohort is biased in European ancestry [29].

### Biotypes show opposite methylation patterns in dopaminergic and serotonergic pathways

**Fig. 6:**
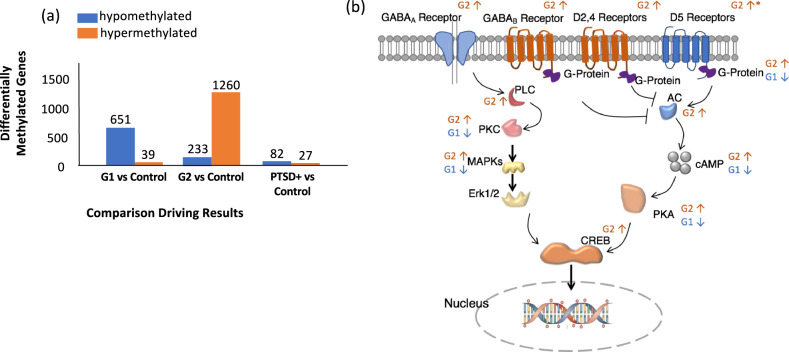
The biotypes oppositely regulated in dopaminergic and serotonergic pathways. **a** Comparison of the differentially methylated genes (DMG) of G1 versus control, and G2 versus control, and the combined PTSD+ group (G1 and G2) versus control, from the combined Discovery and Replication cohorts. The orange and blue bars indicate the number of hyper-/hypo-methylated genes, respectively. **b** The differentially methylated pathways, identified from the analysis of the DMGs, showed overlap between the subtypes in the Dopamine-cAMP-PKA-CREB and GABA-PKC-CREB signaling pathway. *Only the probe for DRD5 is located in the promoter region. For all others, an upward arrow indicates hypermethylation corresponding to activation, and a downward arrow to hypomethylation, corresponding to suppression.

By combining the Discovery and Replication cohorts, the average *β*-values between the groups after adjusting for cell compositions, ancestry (first three principal components (PCs) from Genome-Wide Association Studies (GWAS)), and age was examined. A total of 2039 probes (1493 genes) were significantly differentially methylated between the G2 biotype and the healthy biotype (*p* < 0.01). 1260 of 1493 differentially methylated genes (DMGs) were hypermethylated in the G2 biotype, while 651 of 690 DMGs were hypomethylated in the G1 biotype. Because of the opposite methylation patterns of the two biotypes of PTSD, most of these DMGs mathematically averaged out the difference over control when considering all PTSD as one group. Such a comparison resulted in merely 109 DMGs (Fig. 6a) in the aggregate PTSD group compared to controls. There were only 38 DMGs overlaps in two categories and regulated in the same direction.

For the significantly enriched pathways (Table S5), all the G1 enriched pathways were downregulated, and those of G2 were upregulated. Using hierarchical clustering to recreate convergent functional groups (Fig. S3), we discovered that Dopamine-PKA-CREB and GABA-PKC-CREB signaling pathways are the central cascades commonly dysregulated in both biotypes. As illustrated in Fig. 6b, multiple dopamine and GABA receptors showed greater methylation in G2 and cascaded to CREB via both PKA and PKC signaling pathways. In contrast, G1 showed less methylation in PKA/PKC pathways.

### Biotyping improves diagnostic markers

As aforementioned, the G1 biotype is similar to controls in methylation pattern, and hereby reduces the sensitivity of PTSD diagnostic biomarkers. Comparing two biotypes on a 28-multi-omics marker panel proposed by Dean et al., the prediction sensitivity based on an LSVM classifier for the G1 vs. control and G2 vs. control were strikingly different (sensitivity: 0.36 vs. 0.93, area under the ROC curve (AUC): 0.5 vs. 0.89). Notably, even the epigenetic markers from this panel were removed, similar AUC values of 0.49 and 0.85 were observed for G2 and G1, respectively (Table 1).

**Table 1:** Comparison of classification performance between two biotypes based using independently identified diagnostic biomarker panels.

	G1 (11) vs. Control (26)	G2 (15) vs. Control (26)	PTSD + (26) vs. Control (26)
Dean’s multi-panel biomarkers* [28]			
AUC	0.50	0.89	0.71
Sensitivity	0.36	0.93	0.69
Specificity	0.81	0.77	0.69
Dean’s multi-panel biomarkers less epigenetic markers*			
AUC	0.49	0.85	0.72
Sensitivity	0.63	0.96	0.81
Specificity	0.50	0.60	0.65

*A LSVM classifier was trained on Dean’s 28 mixed panel [28] in Discovery, and compared the classification performance for G1 versus control, G2 versus control, and all PTSD+ versus control from the Replication cohort. The numbers denoted in parentheses are the *N* for each cohort. The classification performance is measured by AUC (area under the curve), as well as sensitivity and specificity defined by Youden index. A similar comparison was repeated when epigenetic markers removed (17 mixed biomarkers).

To further prove that this biomarker is not an exception, a 26-gene epigenetic marker was independently developed using a conventional random-sampling-based feature selection (see Material and method) without prior knowledge of biotypes. The performance of the G1 type and the G2 type is similar to Dean’s marker using the LSVM classifier (sensitivity: 0.55 vs. 0.93, AUC: 0.67 vs. 0.85) with overall AUC 0.77. This panel can also separate PTSD/MDD comorbidity and MDD alone. If the individuals with the BDI cutoff larger than 10 are considered as MDD, it results in 8 MDD alone and 43 PTSD/MDD in combined Replication and Follow-up cohorts. The same LSVM classifier was applied to distinguish both MDD vs. PTSD/MDD in the combined cohort. The error rate is 13.7% for MDD vs. PTSD/MDD, higher than 16.8% for PTSD/non-PTSD case.

However, when the biotypes are considered (see Method), and it resulted in a 12-gene panel (Table S6) selected from the 26-gene panel. The overall performance has been improved to 85% sensitivity, 82% specificity, and AUC 0.85 (Table 2). And the performance is fairly stable across four classification methods, including LSVM, Random Forest, LDA, and polynomial SVM (Table S7).

**Table 2:** Comparison of classification performance between two biotypes towards improvement in a diagnostic biomarker panel.

	G1 (11) vs. Control (28)	G2 (15) vs. Control (28)	PTSD + (26) vs. Control (28)
26 DNAm markers*			
AUC	0.67	0.85	0.77
Sensitivity	0.55	0.93	0.81
Specificity	0.82	0.71	0.71
12 DNAm markers*			
AUC	0.78	0.89	0.85
Sensitivity	0.73	0.93	0.85
Specificity	0.86	0.82	0.82

*An LSVM classifier was trained newly identified 26-gene panel and refined 12-gene panel in Discovery, and compared the classification performance for G1 versus control, G2 versus control, and all PTSD+ versus control from the Replication cohort. AUC (area under the curve), as well as sensitivity and specificity are reported.

Note only one of the 12 genes overlapped with the 100 biotyping genes, and the predicted PTSD probability of 12-gene PTSD biomarker did not significantly associate with PTSD severity (*r* = 0.33, *p* = 0.100). In this sense, the 100-gene biotyping panel and 12-gene PTSD diagnostic panel are complementary tools to assess PTSD risk.

## Discussion

To our knowledge, this is the first attempt of identifying and validating epigenetic biotypes in psychological disorders. Two biotypes that were computationally derived showed a stronger association with PTSD risk, and were successfully validated in the 3-year follow-up, two independent replication cohorts, and an active duty longitudinal cohort. The major findings in this study were: (1) The biotypes differentiate in the PTSD risk with evidence in PTSD/MDD comorbidity, PRS, clinical anxiety, and depressive symptoms. (2) The G2 biotype consistently has higher clinician ratings than G1 in all replication cohorts, including active duty cohort (3) The G1 biotype generally recovers faster than G2 in veterans and active duty, while the G2 biotype may be associated with an increased PTSD risk in healthy veterans. (4) The biotyping algorithm switches the biotypes accordingly, when the individuals significantly change their PTSD severity in the follow-up. (5) Compared to healthy controls, the G2 biotype has greater methylation that is expected to result from risk factors such as higher stress exposure, while G1 has less methylation than controls. (6) The biotypes show opposite methylation patterns in dopaminergic and serotonergic pathways that associate with anxiety and depressive symptoms. (7) A high-performance PTSD diagnostic marker was proposed as an application of biotypes. (8) The biotypes are preserved in active duty military personnel, and show potential applicability as a screening tool in non-clinician-administered settings.

The subtyping takes advantage of a more expanded set of idiosyncratic symptoms and functional ratings than the use of PTSD symptom severity cut off per se. The heterogeneity of many variables that move in tandem with PTSD and its comorbidities characterize new phenotypes among the PTSD cases. Using severity cut off, a 26-gene panel can be identified to distinguish PTSD from the healthy phenotype. But this panel has a weaker association with the CAPS score in PTSD cases than the biotypes (*r* ~0.3 vs. 0.5 in the Replication cohort). Moreover, the current cohort revealed two biotypes related to anxiety and depressive symptoms, but it is possible, and perhaps probable, that with a larger and distinct population we may identify additional biotypes related to dissociation and others.

Among a long list of clinical symptoms and functional ratings, the biotypes in this study are largely different in anxiety, depressive symptoms, sleep disturbance, and BP. The PTSD/MDD comorbidity is highly enriched in the G2 biotype, which also has elevated PRS. Furthermore, the underlying biological pathways enriched when comparing the two biotypes and healthy controls oppositely regulated, in the Dopamine- -PKA-CREB and GABA-PKC-CREB signaling pathways. Greater methylation in dopaminergic and serotonergic pathways can lead to higher anxiety and depressive symptoms [39, 40] in major depression [41] and schizophrenia [42]. In blood cells, CREB is a powerful transcription factor associated with Type II diabetes [43], and cognitive dysfunction [44]. The altered regulation of these pathways may be the biological explanation for the differences in clinical symptoms between the two biotypes, and the usages of blood pressure medicines, pain killers, antidepressants, and sleep pills. Altogether, the underlying molecular difference paired with specific clinical features may provide useful guidance towards personalized treatment for PTSD.

In the active duty longitudinal study (Fort Campbell Cohort), around 50% of individuals from the previous time-points were retested. Many participants have been deployed multiple times, and thus some soldiers already had high PCL scores at pre-deployment, but most of them did not return to test in phase 2 and 3. Immediately after-deployment reported lower symptoms, and have less difference in biotypes than the latter time-point. For the 473 soldiers who completed testing for all three time-points, there is an “increasing” trajectory (*N* = 43, 9.1%) and a “resilient” trajectory (*N* = 430, 90.9%) of PCL-5 scores [45]. Multiple factors may be related to lower reported symptoms immediately after deployment than a few months after deployment, such as attrition and reluctance to report. There are few studies examining veterans immediately after deployment, and the natural course of PTSD including peaks and valleys of symptom severity remains unknown.

Our study has several strengths. This is the first study to identify and validate epigenetic biotypes in veteran and active duty personnel. The current findings demonstrate that the epigenetic profiles of blood cells in response to varying stress-induced injury can create potential to assess PTSD risk following warzone trauma. The discovery cohort contained well-phenotyped male veterans who were all exposed to combat trauma, while the matching control group also exposed to combat-related trauma never developed PTSD, helping tease out the impact of PTSD versus the experience of trauma per se. Multiple replication cohorts have been used to validate biotypes including a 3-year follow-up, two independent veteran replications, and an active duty cohort. These consistent repeats greatly improve the reliability of the biotypes despite the remarkable complexity that exists in self-reports, study population, and high-throughput assays. As another strength, the subtyping was applied on an MDD alone cohort, helping to tease out the impact of depression comorbidity. Finally, our approach integrates many idiosyncratic clinical-features, to reduce the influence of technical artifacts in high-throughput DNAm arrays [46] and achieve clinical-relevant biotypes, comparing to the approaches based on the omics alone, such as unsupervised clustering.

Limitations of our study include the following: (1) our cohort focus on only males and only combat-related PTSD, especially those that have CAPS scores greater than 40, limiting generalizability to other populations; (2) the longitudinal data only included two discrete points in time, separated by ~3 years apart; (3) our cohort does not contain a healthy control group without trauma exposure; (4) the sample size of our Replication and Follow-up cohort were relatively small, thus requiring replication in the future using larger samples; (5) Some boundary cases may switch to another type in technical repeats due to experimental variation in high-throughput assays.

Overall, our findings link objective biological measures with clinical phenotypes in PTSD and may provide useful guidance towards personalized treatment for PTSD. First, this study proposes a high-performance 12-gene methylation panel to help the clinicians diagnose PTSD cases using the non-biased molecular assay. Second, the biotyping method can be a screening tool to subtype active duty personnel with probable PTSD symptoms in non-clinician-administered settings. These potential applications are promising in treatment-matching and monitoring of clinical outcomes.

## Conclusions

Using a variety of computational strategies, PTSD biotypes were identified that show distinct methylation and clinical features strongly associated with PTSD symptomatic severity, especially hyperarousal, which may be explained by a subset of defined molecular pathways. This subtyping can provide useful guidance to improve diagnosis, decrease variance in understanding PTSD cohorts, understand underlying pathologic and susceptibility mechanisms, and contribute to the development of personalized therapeutic options for distinct groups of patients. Furthermore, this work may have applicability in screening active duty soldiers in the field.

## Material and methods

### Study population

The protocols for all studied cohorts were approved by the respective Institutional Review Board at each study site and Human Research Protection Office from the Department of Defense.

#### PTSD Systems Biology Consortium cohorts

Data from combat trauma-exposed male veterans with and without PTSD collected in association with three separate studies (grants W911NF-13-1-0376, W911NF-17-2-0086, W81XWH-09-2-0044, and W81XWH-14-1-0043) were reanalyzed for the purpose of obtaining biotypes. All veterans in the cohorts served in Operation Iraqi Freedom/Operation Enduring Freedom (OIF/OEF). The “Discovery” cohort (83 PTSD positive and 83 PTSD negative), the “Follow-up” cohort (3-year follow-up of a subset of the “Discovery” cohort: 16 PTSD positive, 12 subthreshold PTSD and 31 PTSD negative), and an independent combat trauma-exposed “Replication” cohort (26 PTSD positive and 28 PTSD negative).

#### Inclusion/Exclusion

All participants (including the PTSD negative controls) were exposed to war-zone-related stressors, and no significant age or ethnicity difference was found across PTSD positive and negative groups (Table 3). Veterans with PTSD had warzone-related PTSD symptoms for at least a 3-month duration with a current CAPS total score ≥40. The comparison group consisted of veterans who had also been exposed to warzone stressors and had a past-month CAPS total score ≤20 and did not meet the lifetime criteria regarding the previous diagnosis with PTSD.

**Table 3:** Summary of veteran, active duty, and civilian cohorts.

	Veteran													Active duty			Civilian		
	Discovery			Follow-up				Replication			Bronx VA			Ft Campbell			UCSF		
Variables	PTSD+(83)	PTSD−(83)	*p* value *T* test	PTSD+(16)	PTSD Sub (12)	PTSD −(31)	*p* value ANOVA	PTSD+(26)	PTSD−(28)	*p* value	PTSD+(28)	PTSD−(10)	*p* value	PTSD+(76)	PTSD Sub (57)	*p* value	MDD+	MDD−	*p* value
BMI (SD)	29.78 (6.13)	28.42 (4.80)	0.118	31.99 (5.16)	30.44 (7.96)	28.29 (4.17)	0.085	29.45 (6.71)	23.75 (9.06)	0.014	30.94 (4.64)	29.54 (4.31)	0.407	27.30 (3.68)	28.71 (3.35)	0.023			
Age (SD)	32.68 (7.34)	32.52 (8.03)	0.901	33.63 (7.49)	34.92 (7.99)	35.97 (8.45)	0.643	36.46 (10.6)	33.79 (9.26)	0.331	36.88 (8.54)	35.9 (11.76)	0.815	28.74 (5.90)	28.12 (5.49)	0.538	40.47 (15.4)	38.68 (13.7)	0.504
Neutrophil % (SD)	56.09 (9.16)	54.45 (9.81)	0.282	50.63 (11.36)	51.95 (7.38)	52.70 (12.04)	0.840	57.21 (10.05)	58.17 (8.89)	0.739	–	–	–	–	–	–			
Lymphocyte % (SD)	33.07 (8.11)	34.59 (9.45)	0.271	37.97 (10.53)	36.52 (6.72)	34.73 (10.60)	0.577	31.29 (9.44)	29.2 (7.83)	0.429	33.49 (10.76)	38.17 (9.78)	0.140	–	–	–			
Monocyte % (SD)	7.78 (2.09)	7.93 (1.98)	0.651	7.57 (1.39)	7.75 (1.95)	8.83 (2.20)	0.084	8.06 (2.03)	8.65 (2.98)	0.448	8.19 (1.95)	7.35 (1.78)	0.157	–	–	–			
Eosinophil % (SD)	2.60 (1.54)	2.58 (1.49)	0.922	3.09 (1.61)	3.14 (2.82)	3.16 (2.27)	0.994	2.72 (2.57)	3.48 (1.86)	0.263	–	–	–	–	–	–			
Basophil % (SD)	0.51 (0.26)	0.59 (0.35)	0.117	0.53 (0.37)	0.47 (0.18)	0.54 (0.23)	0.771	0.51 (0.30)	0.48 (0.17)	0.706	–	–	–	–	–	–			
CAPS total current (SD)	68.65 (16.91)	3.78 (5.14)	<0.001	70.88 (18.75)	36.50 (9.43)	4.87 (7.13)	<0.001	67.15 (19.37)	3.57 (5.93)	<0.001	84.76 (12.77)	7.4 (6.54)	<0.001	–	–	–			
PCL score (SD)	61.24 (11.84)	25.93 (8.98)	<0.001	61.54 (9.96)	44.45 (10.29)	26.57 (13.81)	<0.001	53.43 (14.93)	22.69 (7.31)	<0.001	–	–	–	48.49 (8.67)	33.72 (2.26)	<0.001			
BDI score (SD)	25.06 (10.82)	5.83 (6.23)	<0.001	25.67 (12.36)	18.83 (10.50)	3.63 (4.94)	<0.001	22.47 (11.48)	7.11 (8.40)	<0.001									
Smokers*	21	8	<0.001*	5	5	5	<0.001*	17	6	<0.001*									
Alcohol Use Identification Test (SD)	3.22 (3.69)	2.92 (2.42)	0.531	2.31 (4.40)	2.09 (1.87)	2.97 (2.80)	0.667	3.74 (3.77)	3.20 (2.53)	0.568									
Numbers of deployment	1.82 (0.84)	1.77 (0.89)	0.720	1.93 (1.07)	1.82 (0.87)	1.65 (0.75)	0.651	1.35 (0.56)	2.07 (1.46)	0.020									

Veterans with PTSD positive had warzone-related PTSD symptoms for at least a 3-month duration with a current CAPS total score ≥40. The PTSD negative veterans who had also been exposed to warzone stressors with a current CAPS total score ≤20 and did not have lifetime PTSD. The veterans with a current CAPS total score between 20 and 40 were PTSD subthreshold.

Exclusion criteria: history of moderate to severe traumatic brain injury; drug abuse within the past year or alcohol dependence within the past 8 months; prominent suicidal or homicidal ideation; lifetime history of any psychiatric disorder with psychotic features, bipolar disorder, obsessive-compulsive disorder; neurologic disorders affecting central nervous system function; and subjects who were not stable for more than 2 months on psychiatric medication, anticonvulsants, antihypertensive medication or sympathomimetic medication. Comorbid MDD (BDI ≥ 18) were not exclusionary if PTSD was considered the primary diagnosis.

Bronx VA cohort also excluded the presence of diabetes mellitus or any current unstable medical condition that represents a contraindication to taking glucocorticoids.

Active duty exposed to warzone stressors with PCL5 ≥ 38 were defined as PTSD positive, the individuals with PCL5 < 28 were PTSD negative. The others were PTSD subthreshold.

*This *p* value is based on Chi-square test.

The Follow-up cohort consisted of veterans from the Discovery sample who agreed to return 3 years later for a follow-up blood draw and clinical assessment. Veterans were included regardless of CAPS scores (e.g., they could present with subthreshold PTSD including “high subthreshold” if their current CAPS total scores were ≥40, and “low subthreshold” with current CAPS total scores between 20 and 40).

For all participants, those with the following comorbidities were excluded: a history of moderate to severe traumatic brain injury; drug abuse within the past year or alcohol dependence within the past 8 months; prominent suicidal or homicidal ideation; lifetime history of any psychiatric disorder with psychotic features, bipolar disorder, obsessive-compulsive disorder; or neurologic disorders affecting central nervous system function; and subjects who were not stable for more than 2 months on psychiatric medication, anticonvulsants, antihypertensive medication or sympathomimetic medication. Comorbid MDD was not exclusionary if PTSD was considered the primary diagnosis.

#### Clinical phenotyping

The participants in the OIF/OEF veteran cohorts were evaluated by a licensed clinical psychologist with clinician-administered DSM-4 and structured clinical interview for the DSM (SCID) [47] interviews, and diagnoses confirmed with a consensus conference. Participants completed several well established PTSD measures (CAPS, PCL [48], and MSC), Peri-traumatic Dissociative Experiences Questionnaire (PDEQ), general psychiatric symptoms (SCL90 scores, BDI, etc.) and health conditions (12-item Short Form Health Survey (SF12)) [49], PSQI, early trauma experience (ETI score), Alcohol Use Identification Test (AUDIT) [50], stress level (Perceived Stress Scale (PSS)) [51], and other measurements. All clinicians who conducted the clinical interviews for this study were doctoral-level psychologists who had several years of experience working with veterans and civilian trauma victims. PTSD diagnoses were calibrated across sites in a weekly meeting to ensure the consistent application of measures.

#### Veteran Bronx VA cohort

The second replication cohort (28 PTSD positive and 10 PTSD negative) contains the pre-treatment subjects from an oral hydrocortisone study (grants W81XWH-10-2-0072 and W81XWH-13-1-0071) conducted at the Icahn School of Medicine at Mount Sinai. The subjects were recruited from the James J Peters Veterans Affairs Medical Centers in Bronx, New York. Subjects were similarly diagnosed using the DSM-4 diagnosis, SCID interview, and CAPS scores with the same exclusion criteria as the OIF/OEF veteran cohorts.

#### Active duty Fort Campbell cohort

The third replication cohort was an active duty longitudinal cohort. The participants in this cohort were recruited from Fort Campbell, Kentucky, and assessed before and after a 10-month deployment to Afghanistan in 2014 (granted by Steven A. and Alexandra M. Cohen Foundation, Inc. and Cohen Veterans Bioscience, Inc. (CVB)). Phase I assessment occurred 2-weeks prior to deployment, while Phases II occurred 3-days after returning from a 10-month tour of duty, and Phase III occurred 90–180 days post-deployment. Subjects were diagnosed in this cohort using the self-report PCL-5 [52]. To approximately match the PTSD severity of the veteran cohorts noted (CAPS ≥ 40) previously, PTSD-positive individuals were defined as those with PCL ≥ 38 with trauma exposure (Phase I: *N* = 10, Phase II: *N* = 21, and Phase III: *N* = 45) and PTSD subthreshold with 38 > PCL ≥ 28 (Phase I: *N* = 11, Phase II: *N* = 11, and Phase III: *N* = 57). The Phase I PTSD positive had pre-existing PTSD before current deployment. The PCL-5 cutoff was suggested to be either 33 [53, 54] or 38 [55] for veterans being screened for symptoms of PTSD. The higher PCL-5 cutoff was chosen for moderate and more severe PTSD to equate CAPS total score 40 in CAPS-IV manual [56].

#### MDD UCSF cohort

The fourth cohort was a civilian MDD study collected by the University of California, San Francisco under clinical trials NCT00812994 and NCT00285935 (49 MDD/63 Healthy Control). Depressed participants were diagnosed with a current major depressive episode, without psychotic features, with the Structured Clinical Interview for DSM IV-TR Axis I Disorders (SCID), and confirmed by the Hamilton Depression Rating Scale (HAM-D) [57] score ≥17. The MDD patients with psychotic symptoms, bipolar disorder, PTSD, eating disorder, recent substance abuse or dependence (including alcohol), chronic inflammatory disorders, neurological disorders, or major medical conditions (e.g., cancer, HIV, diabetes, etc.) were excluded. Comorbid anxiety disorders, with the exception of PTSD, were not exclusionary if MDD was considered the principal diagnosis. In all cases where comorbid anxiety diagnoses existed, both the participant and the psychiatrist concurred that the major reason for participation in the study, the most severe constellation of symptoms and the major cause of concern and disability were the MDD.

### Subtyping approach

#### Clinical features

In the Discovery cohort, we selected 34 clinical features covering PTSD core symptoms (CAPS re-experiencing, avoidance, and hyperarousal reaction, PSQI), Negative Affectivity and psychopathology (e.g., BDI, MSC, SLC90, PCL), Positive Affectivity and Quality of Life (SF-12), as well as peri-traumatic dissociation (PDEQ) (Table S1, Fig. 1a).

#### Epigenetic feature selection

Genome-wide DNAm patterns were profiled using the Infinium HumanMethylation 450 BeadChip (450 K) Kit (Illumina, Inc., San Diego CA, USA). The gold-standard Beta MIxture Quantile normalization method [17] was used to preprocess the beta value. The probes with low standard deviations (sd < 0.05) or extreme values (mean beta < 0.01 or mean beta > 0.99), and intergenic regions were filtered out, and the genes with at least two remaining probes were considered confident (~17,000 probes). To reduce the impact of collinearity and missing values, we identified 1261 highly correlated regions (Pearson’s correlation *r* > 0.8) using the R Igraph package and averaged the methylation values of these regions. Using 41 individuals who did not change diagnostic status at recall, we further filtered out the regions with lower correlation (*r* < 0.65) between original and recall.

Next, we scanned the regions that were modestly associated (|*r*| > 0.2) with one of the 34 clinical features in the Discovery cohort, but not associated with the demographic characters (age, and ethnicity (first three PCs from GWAS)), physiological factors (BMI, cell composition), and factors that are not necessarily related to PTSD severity (smoking status, alcohol usage, and numbers of deployment). It resulted in ~80 regions reflecting with key clinical features for PTSD.

To improve the robustness of the identified regions, we performed a leave-five-out cross-validation test on the Discovery 5000 times using the same pipeline. The cross-validation resulted in 5000 sets of candidate regions, which were ranked by the frequency of appearance in the 5000 sets. We chose a nominal top 100 most frequently appearing gene regions (Table S2) to construct the epigenetic vectors linked with clinical features. The result is similar when choosing the top 80 or 120 (Supporting Note S1). Too few regions can reduce the robustness of the subtyping method.

#### Epigenetic vector construction

CCA is a general form of a multivariate statistical analysis used to explore relationships between two sets of variables, in this case, the 100-gene regions and 34 clinical features measured from the same individuals [58]. Similar to multivariate regression that associates a clinical feature to a list of genes, CCA associates all clinical features and genes simultaneously in order to identify the most correlated orthogonal pairs of gene-clinical composites.

The subject-to-feature ratio was recommended to be less than 20 to stabilize the CCA canonical loadings [59], thus both the 100 regions and 34 clinical features must be reduced to 8 or less considering the 162 subjects in the Discovery cohort. PCA analysis (calculated using R-Swamp package [60]) was used to identify the dominant variation of 100-gene regions that were associated with the clinical features, and the first four PCs of gene regions were enough (Fig. S4). For the 34 clinical variables, the first six PCs explaining 83.6% of the total variance were chosen. After applying CCA to the PCs, we isolated two orthogonal gene-clinical composite pairs with Wilks’ lambda *p* value < 0.0001.

#### PTSD biotype assignment

The subjects of the Discovery cohort were laid out on the coordinates of two DNAm composites, which were paired with different clinical features. An LDA was trained to discriminate between a “control” cluster and a “PTSD” cluster (Fig. 1c). The separation of these two clusters using LDA can be defined by the following equation: −1.06 *x* + 0.15 *y* = 0, whereby *x* and *y* were the two latent DNAm scores. We then assigned the biotype G1 (similar to control, the right side of Fig. 1c), and G2 (distinct to control, the left side of Fig. 1c).

#### PTSD biotype score

To simplify the computation, we define a biotype score as follows,

1 $${\mathrm{Biotype}}\,{\mathrm{score}} = \mathop {\sum }\limits_{i = 1}^{100} w_i \cdot Gene_i$$

Where the weight $$w_i = PCA_{Gene} \cdot CCA_{Gene} \cdot [{\begin{array}{*{20}{c}} { - 1.06} \\ {0.15} \end{array}}]$$; Gene_*i*_ is the methylation profile of a gene region *i*; *PCA*_*Gene*_ is the first four PC loadings of 100-gene regions (a 100 × 4 matrix), and *CCA*_*Gene*_ is the first two unstandardized canonical coefficients of epigenetic features (a 4 × 2 matrix). The weight of each gene was listed in Table S2. The subjects with a biotype score of <0 belong to G2, otherwise G1.

### Differential analysis and pathway analysis

Genome-wide DNAm patterns were profiled using the Infinium HumanMethylation 450 BeadChip (450 K) Kit (Illumina, Inc., San Diego CA, USA). All data analysis was conducted under R version 3.6.0. The association between PTSD status and DNA was conducted utilizing a moderated *t*-test provided by the R limma package v3.40.2 [61] and statistical significance was defined by *p* value < 0.01 and absolute beta value difference >0.02 for cases and controls. The multiple comparison correction FDR < 0.05 resulted in an insufficient number of genes for meaningful pathway analysis, while *p* value < 0.05 resulted in too many false positives, leading to less specific and interpretable enriched pathways. Thus, we compromised *p* value < 0.01, and added a beta value difference cutoff to control the false positives while achieving a reasonable number of genes for pathway enrichment. Ingenuity Pathway Analysis (v 01–08, Qiagen, Redwood City, www.ingenuity.com) was used to determine functional pathway enrichment, which was defined by a *p* value < 0.01 and absolute *z*-score ≥2. The dissimilarity was computed between any two pathways by 1 − |P_*i*_$\cap$*P*_*j*_|/max(|P_*i*_|, |*P*_*j*_|) where |•| is the length of the subset. The hierarchical clustering was applied to the dissimilarity matrix to divide pathways into functional groups using complete linkage.

### PTSD epigenetic diagnostic biomarker identification

The Discovery cohort was used as the training set, and the differentially methylated probes (DMPs) (Limma *t*-test *p* < 0.1 (relaxed for more candidates), absolute median beta difference >0.02) were identified adjusting for cell composition and age. The DMPs were filtered by the Follow-up and Bronx VA cohorts for consistent median beta difference and yielded a list of 77 methylation probes. Next, 100,000 runs of 10-element random sampling were applied, each sampling trained an LSVM classifier on the Discovery, and tested on the Follow-up and Bronx VA cohorts. Once the average AUC was above 0.8, the 10-probe set was retained as a candidate. Finally, the 77-probes were ranked by their frequency in the collection of candidate sets. (Fig. S5a). A forward AUC trajectory was computed to determine the cutoff (Fig. S5b). In the end, the top 29 probes were heuristically selected, while three highly correlated probes were removed to reduce collinearity and resulted in a 26-gene panel (Table S6).

## Disclaimers

The views, opinions, and findings contained in this report are those of the authors and should not be construed as official Department of the Army position, policy, or decision, unless so designated by other official documentation. Citations of commercial organizations or trade names in this report do not constitute an official Department of the Army endorsement or approval of the products or services of these organizations.

## Supplementary information

Figure S1

Figure S2

Figure S3

Figure S4

Figure S5

Legends of supplement figures and tables

Supplemental tables

Supplemental Note S1

Additional consortium members

## Data Availability

All datasets for selected cohorts and code are available with permission through the SysBioCube, at https://sysbiocube-abcc.ncifcrf.gov.
