# Supplementary figures and images for: Epigenetic biotypes of post-traumatic stress disorder in war-zone exposed veteran and active duty males
Source: Mol Psychiatry. 2020 Dec 18;26(8):4300–14. doi: 10.1038/s41380-020-00966-2 (PMC8550967; doi:10.1038/s41380-020-00966-2)

Figure S1

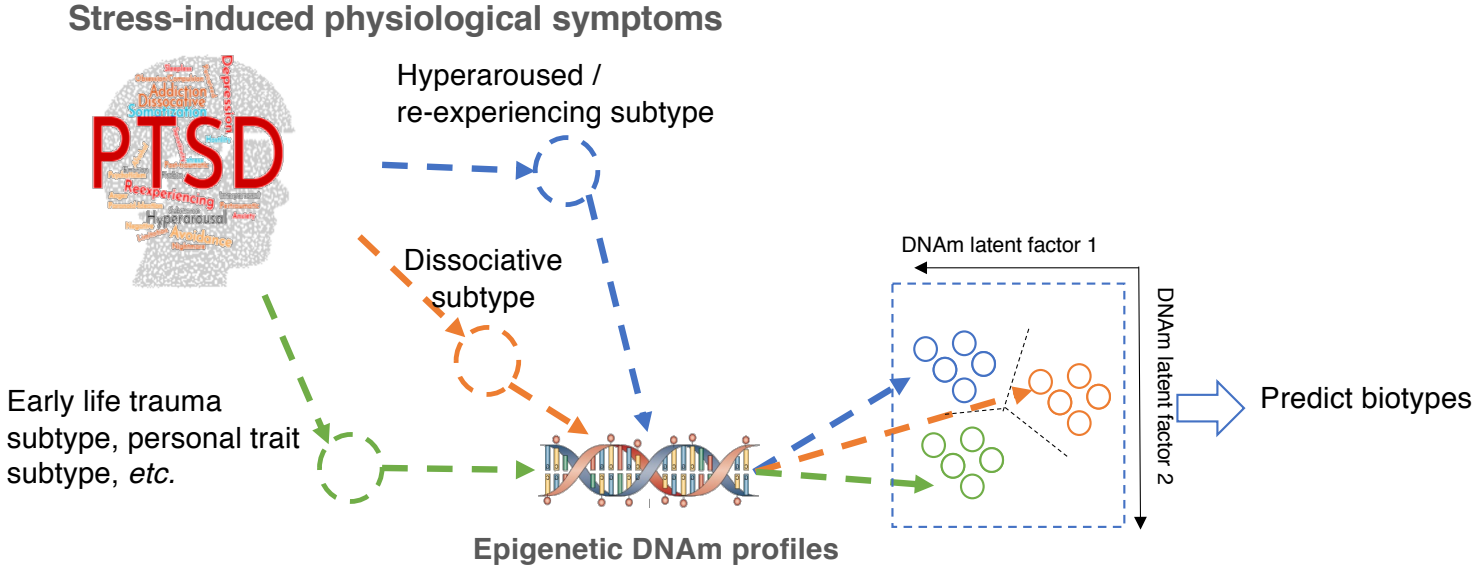

Supplement: Supplementary file 1 — Figure S1 [file 41380_2020_966_MOESM1_ESM.pdf]

Figure S2

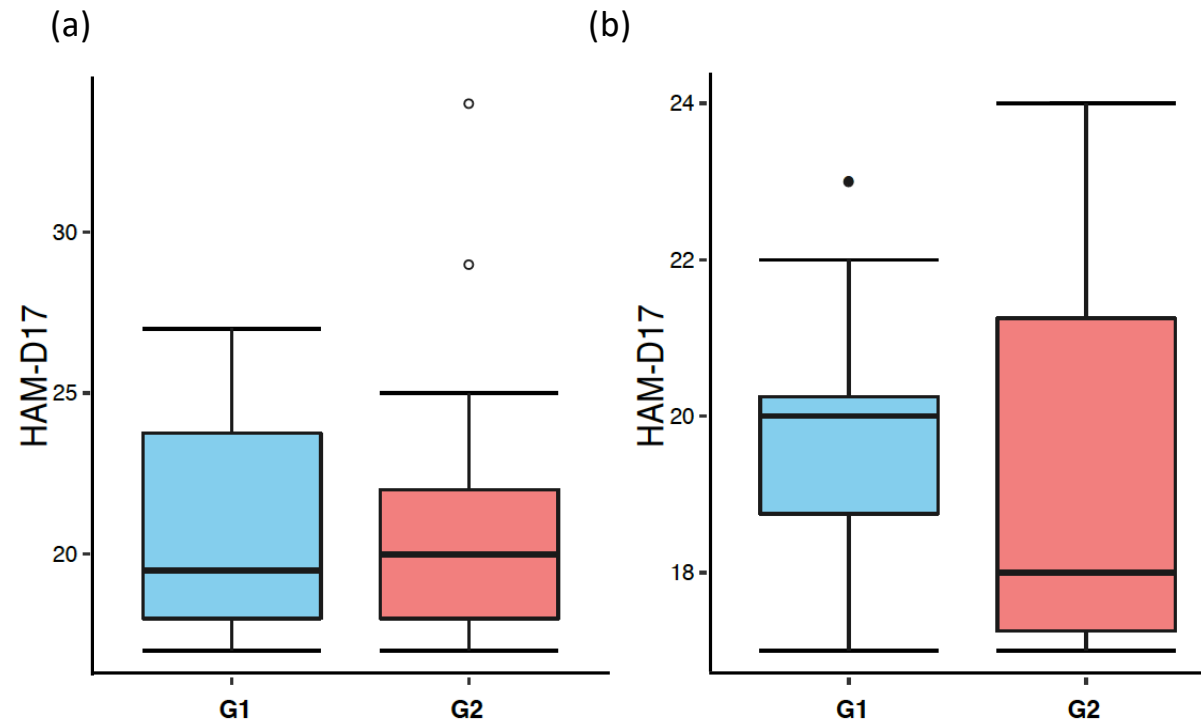

Supplement: Supplementary file 2 — Figure S2 [file 41380_2020_966_MOESM2_ESM.pdf]

Figure S3

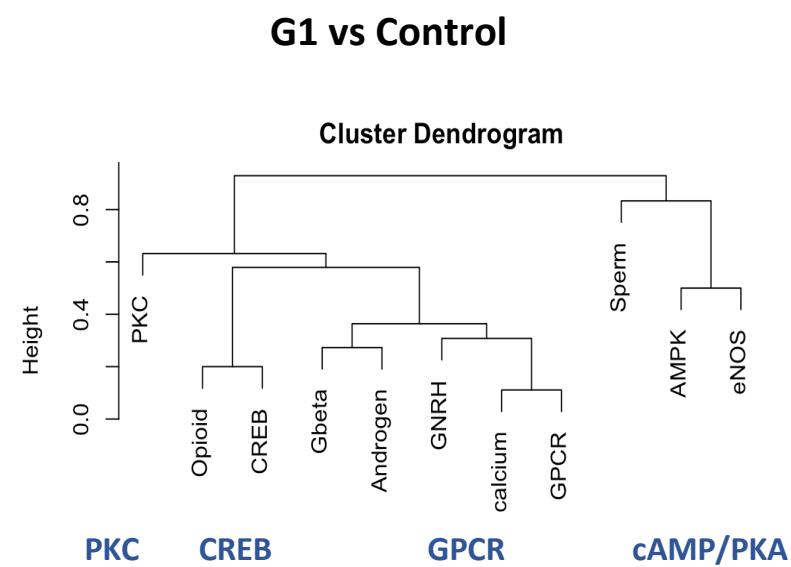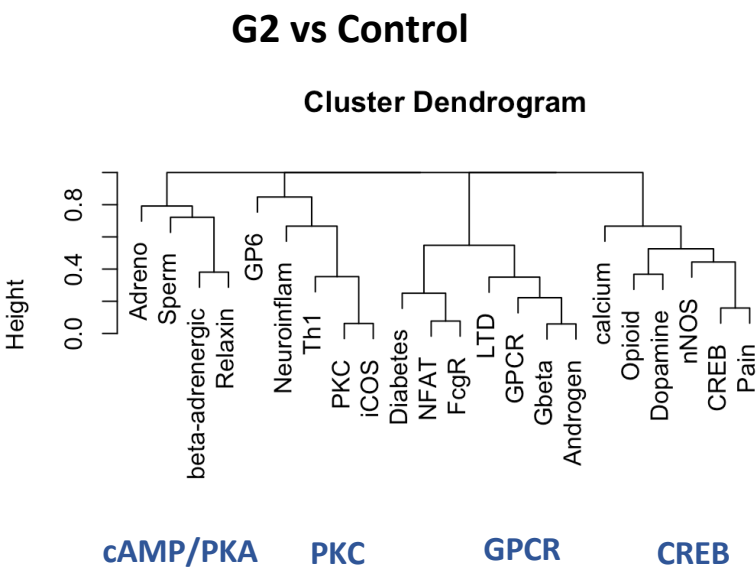

Supplement: Supplementary file 3 — Figure S3 [file 41380_2020_966_MOESM3_ESM.pdf]

**Figure S4**

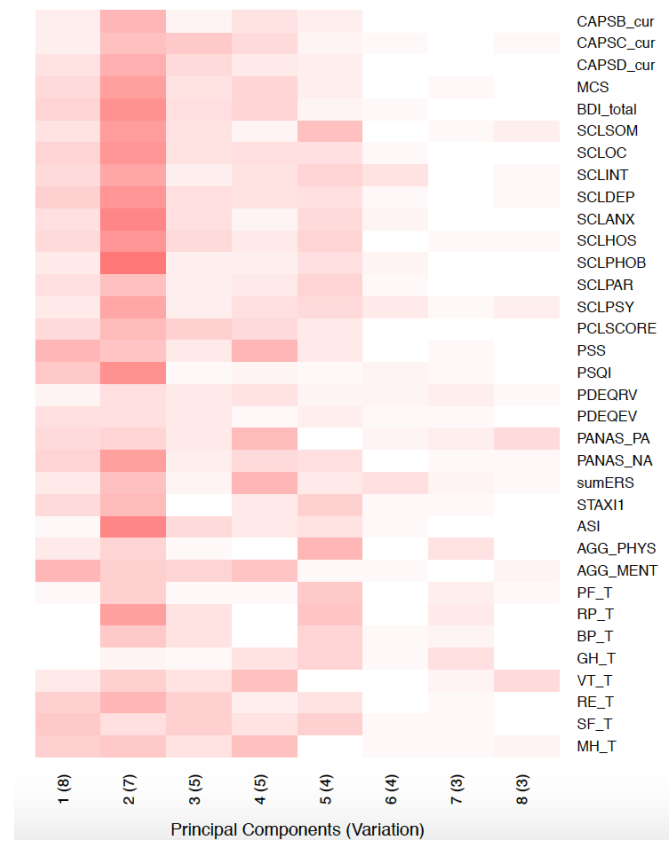

Supplement: Supplementary file 4 — Figure S4 [file 41380_2020_966_MOESM4_ESM.pdf]

Figure S5

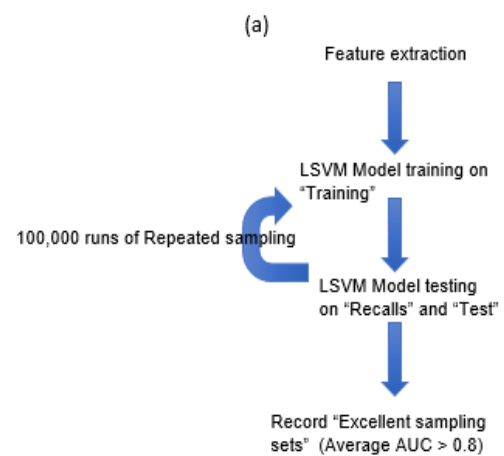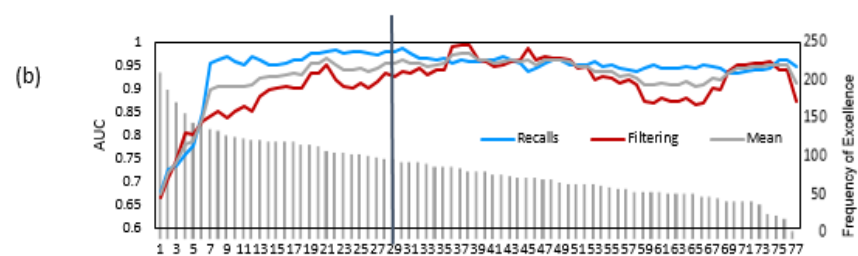

Supplement: Supplementary file 5 — Figure S5 [file 41380_2020_966_MOESM5_ESM.pdf]

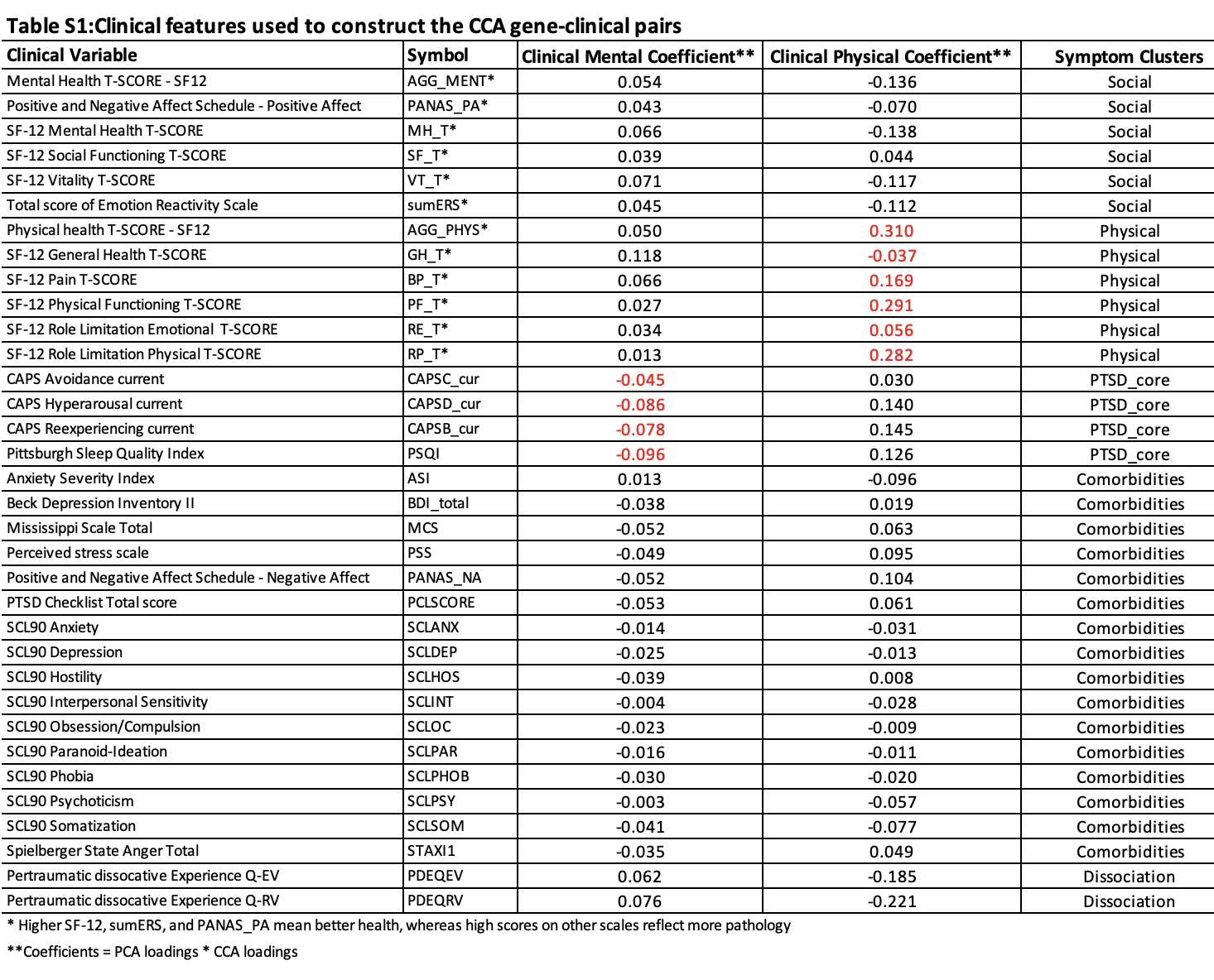


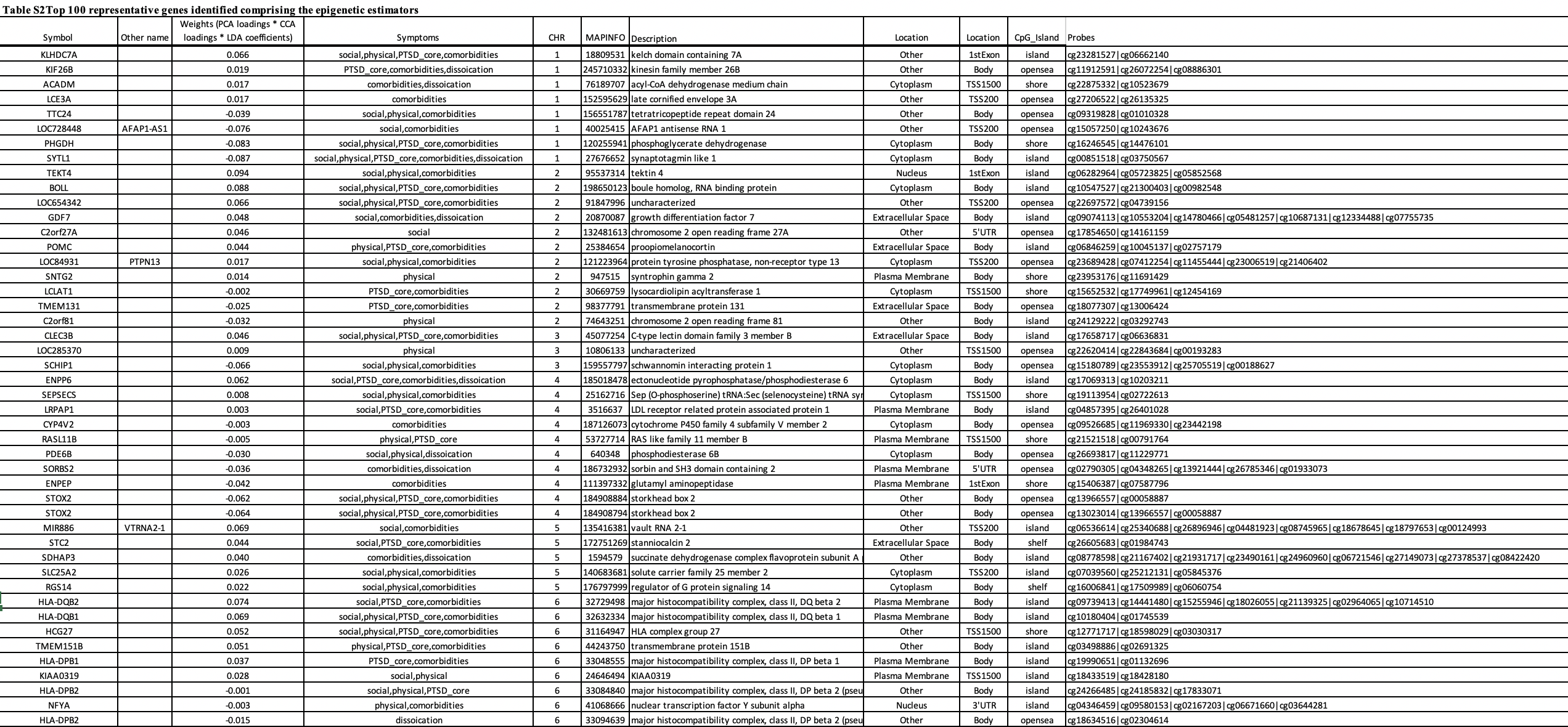


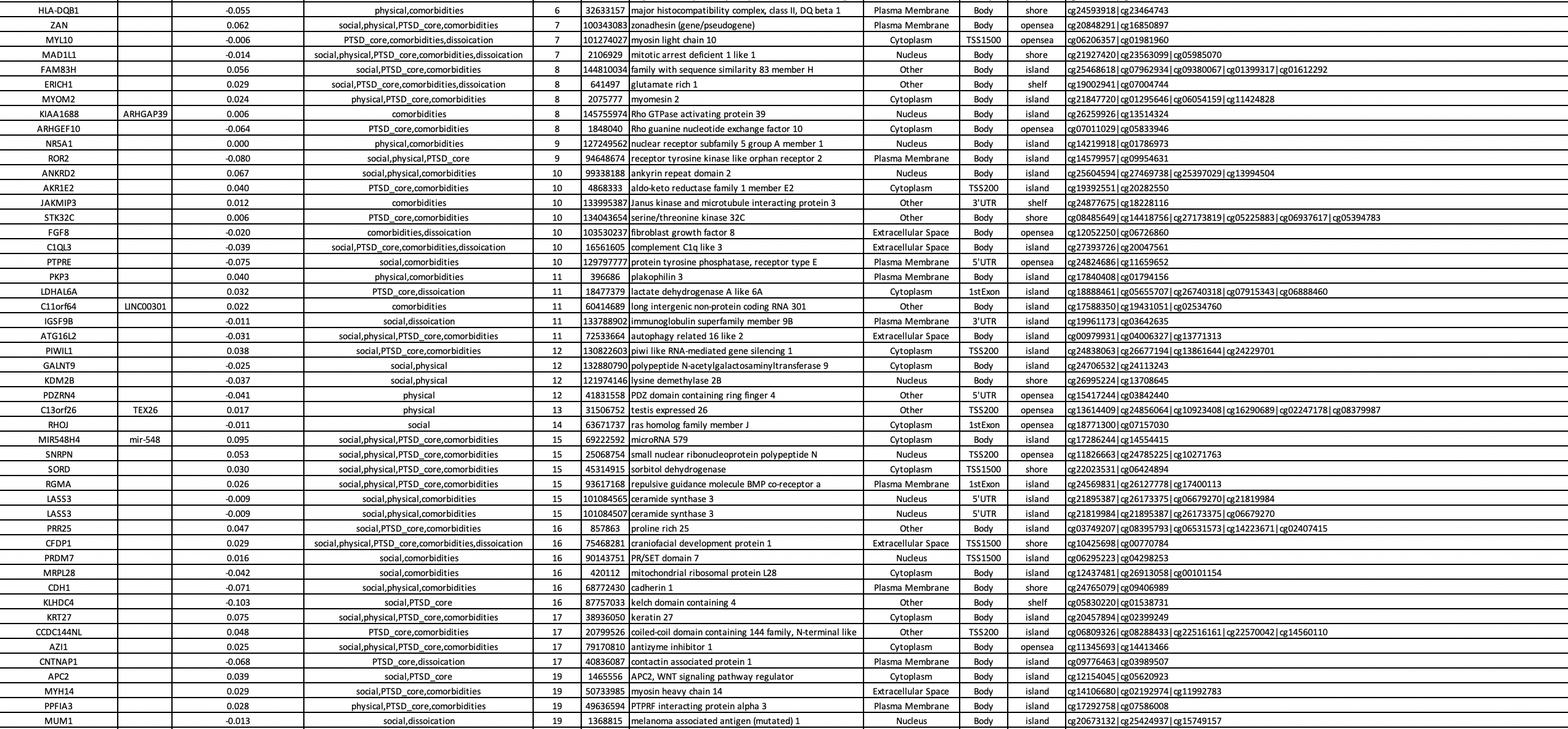


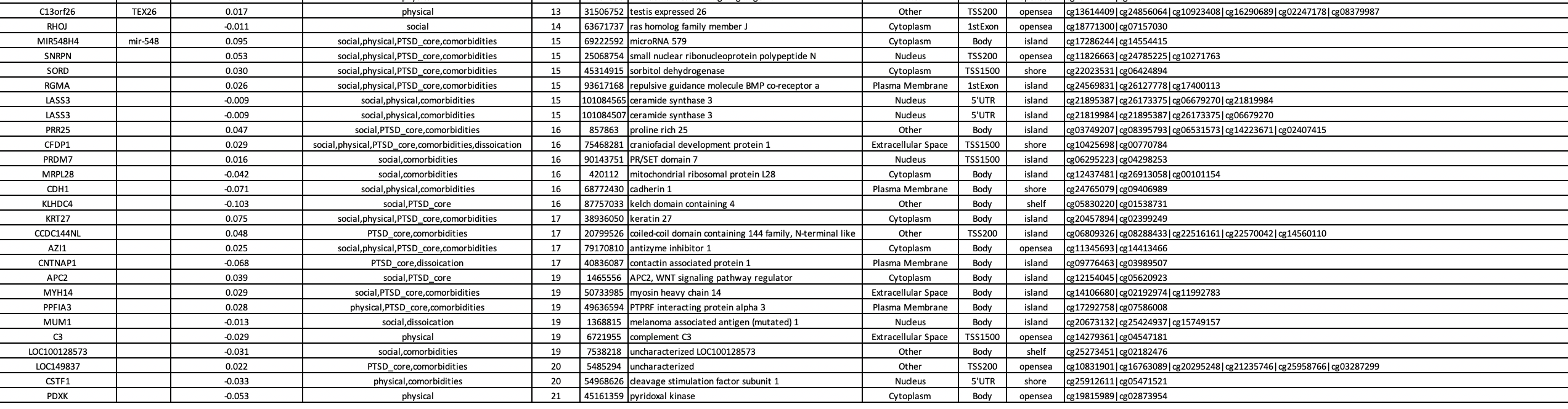


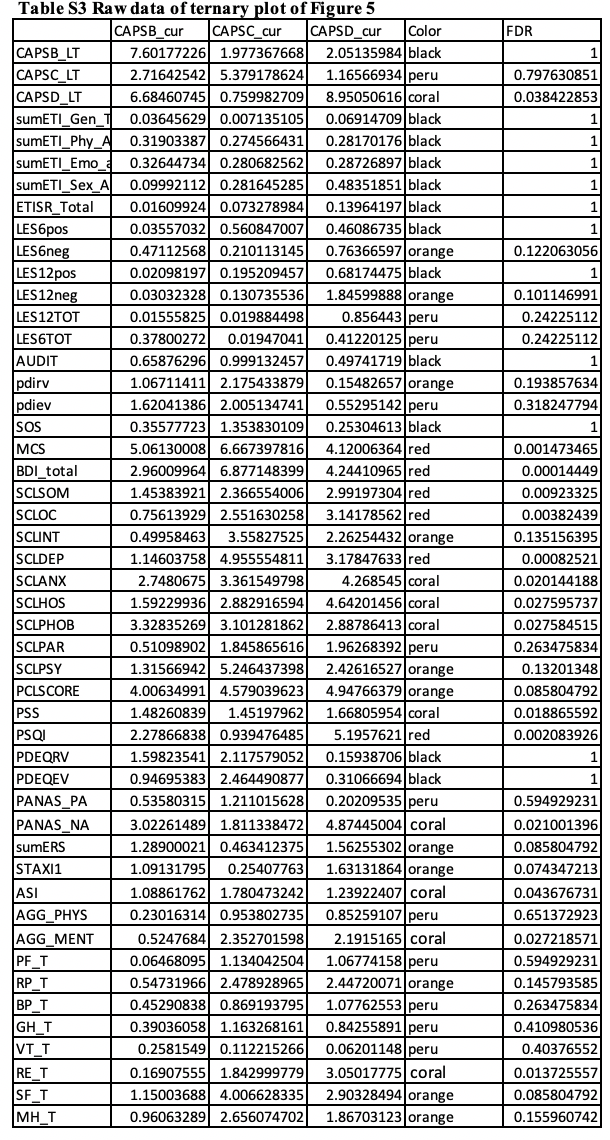


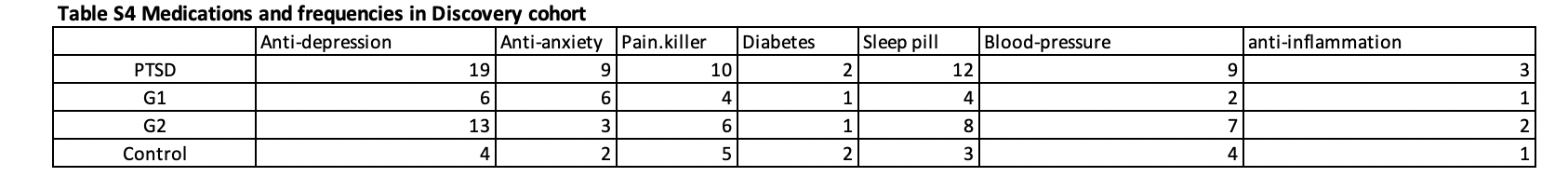


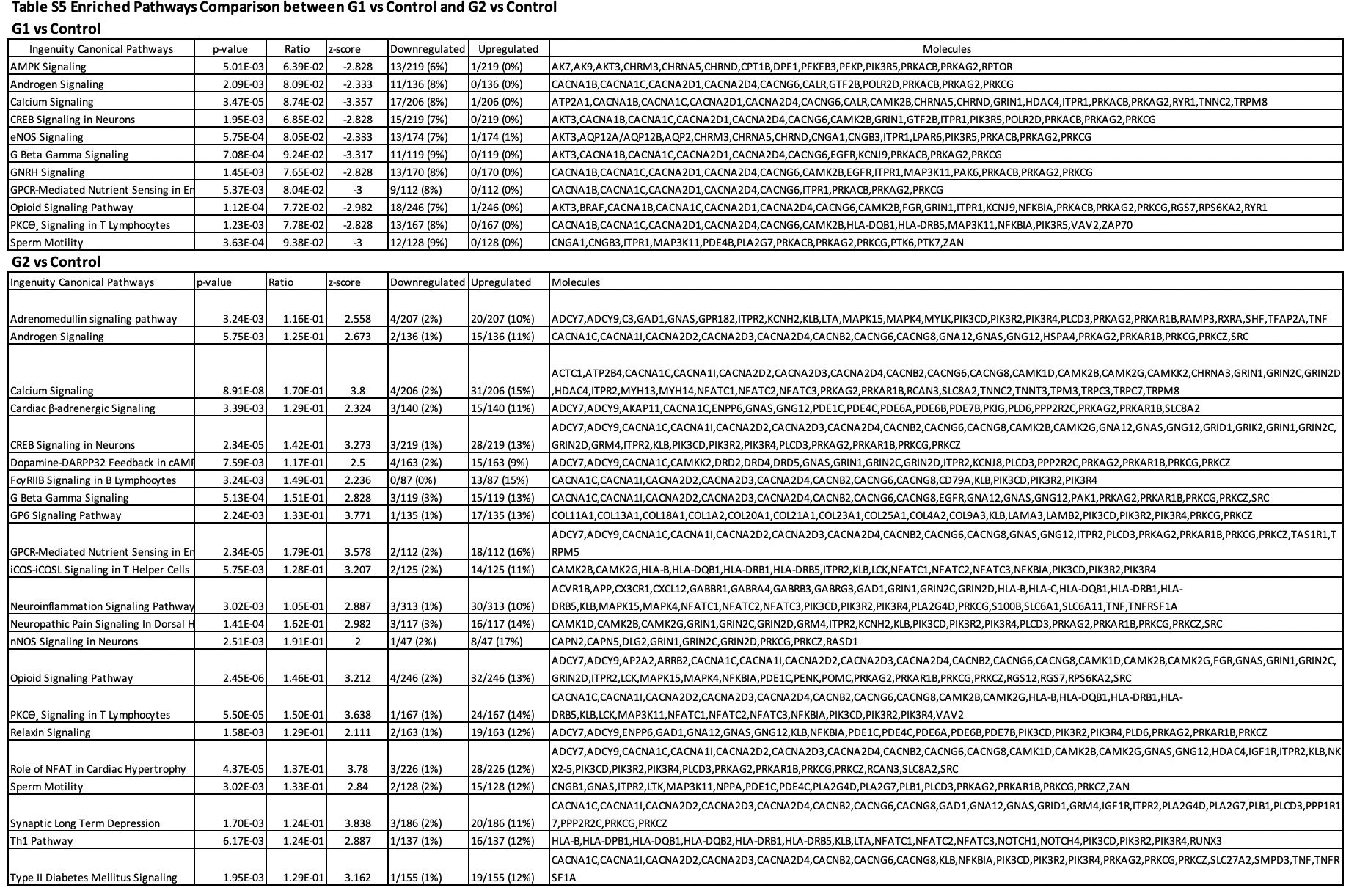


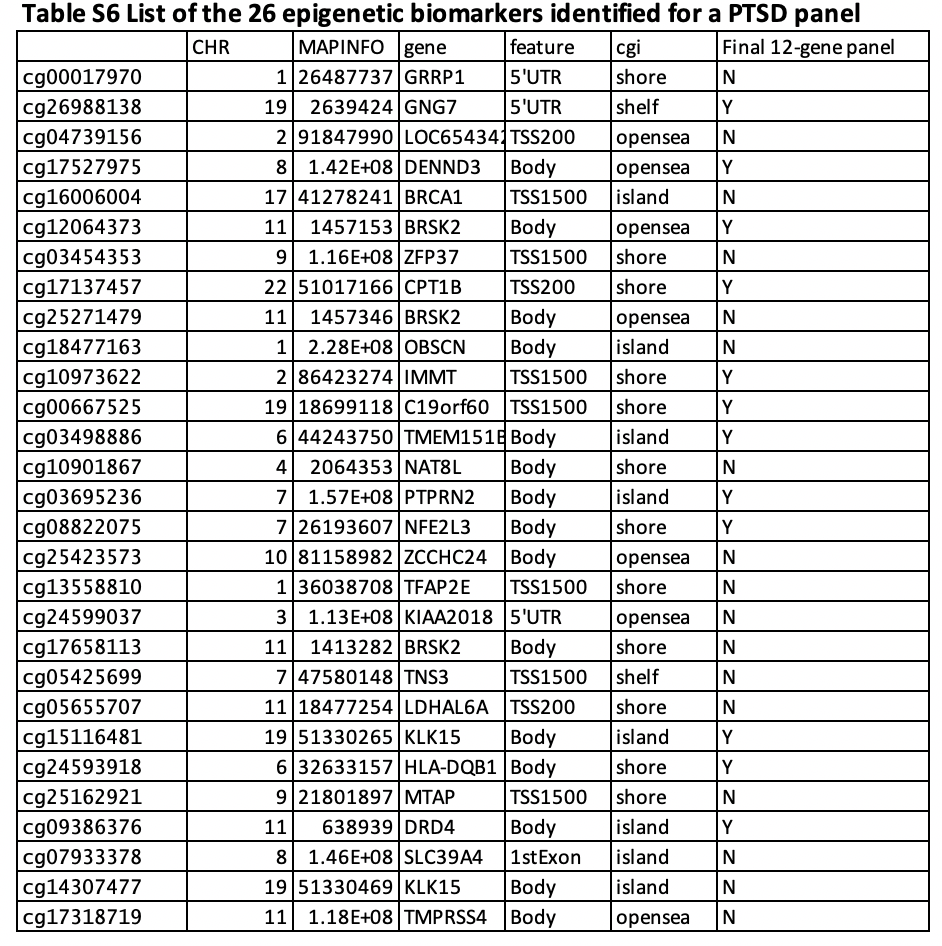


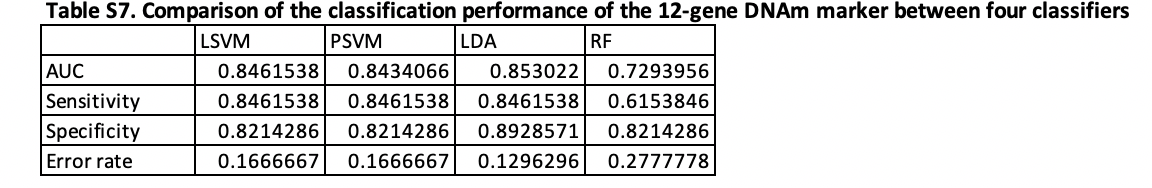

Supplement: Supplementary file 7 — Supplemental tables [file 41380_2020_966_MOESM7_ESM.docx]
