# Supplementary material for: Epigenetic biotypes of post-traumatic stress disorder in war-zone exposed veteran and active duty males
Source: Mol Psychiatry. 2020 Dec 18;26(8):4300–14. doi: 10.1038/s41380-020-00966-2 (PMC8550967; doi:10.1038/s41380-020-00966-2)
Supplement: Supplementary file 6 — Legends of supplement figures and tables [file 41380_2020_966_MOESM6_ESM.docx]

# Supplemental information

**Supporting Materials:**

**Supplemental file S1: Preprocessing, quality control, and Data analysis**

# Supplemental Figures

**Figure S1. DNA methylation can predict biotypes.** Pathological changes associated with different PTSD subtypes result in distinguishable epigenetic patterns (e.g., DNA methylation, DNAm) that can be used to predict biotypes.

**Figure S2. Comparison of G1 and G2 biotypes in an MDD civilian cohort with no PTSD comorbidity.** The depression severity is characterized by 17-item HAM-D. (Male: MDD N =10, control N=17; Female: MDD N =12, control N=10). No statistical difference between biotypes was found.

**Figure S3. Grouping enriched pathways to identify the overlapped signaling pathways.** Enriched pathways were computed on the combined Discovery and Replication cohorts. The hierarchical clustering of enriched pathways based on dissimilarity matrix showed four major clusters, which were named after the actual functions of their pathway names. The height of the dendrogram indicates the degree of difference between branches. The longer the line, the greater the difference. The dissimilarity between pathway *P_i_* and *P_j_* equals 1- |*P_i_*$\bigcap$*P_j_* |/max(|*P_i_*|,|*P_j_*|), where |•| is the length of the subset.

**Figure S4. Theoretical assessment of the number of principal components required for dimension reduction.** The heatmap shows the correlation between the first eight principal components derived from the DNAm profiles of the 100 gene regions and 34 clinical features. The clinical variables (on the vertical axis) are called out using symbols (Note: the symbols are listed in Table S1). The variation explained by each principal component is denoted in parentheses along the X-axis and darker shades indicate higher correlation. Overall, the first four principal components of genes seem to be the most correlated components to clinical features.

**Figure S5.** **(a) Epigenetic biomarker identification pipeline. (b) The trajectory of AUC scores used to identify the 29-biomarker panel.**

# Supplemental Tables

**Table S1. Clinical features used to construct the CCA gene-clinical pairs**

**Table S2. Top 100 representative genes identified comprising the epigenetic estimators**

**Table S3. Raw data of ternary plot of Figure 5**

**Table S4 Medications and frequencies in Discovery cohort**

**Table S5. Enriched pathway comparison between G1 vs Control and G2 vs Control**

**Table S6. List of the 26 epigenetic biomarkers identified for a PTSD panel**

**Table S7. Comparison of the classification performance of the 12-gene DNAm marker between four classifiers**
