# Supplementary material for: Epigenetic biotypes of post-traumatic stress disorder in war-zone exposed veteran and active duty males
Source: Mol Psychiatry. 2020 Dec 18;26(8):4300–14. doi: 10.1038/s41380-020-00966-2 (PMC8550967; doi:10.1038/s41380-020-00966-2)
Supplement: Supplementary file 8 — Supplemental Note S1 [file 41380_2020_966_MOESM8_ESM.docx]

**Blood collection**

*Veteran Discovery, Follow-up, Replication cohort*

Participants reported to the laboratory at James J. Peters VA Medical Center (JJP VAMC) or Icahn School of Medicine at Mount Sinai (ISMMS) at 8:00am after an overnight fast. Vital signs, weight, height and waist-hip ratio were measured and then approximately 160 cc of whole blood was collected and processed for subsequent assays. Blood draws was collected in PAXgene™ DNA tubes (PreAnalytiX GmbH) and frozen until DNA extraction.

*Replication Bronx VA cohort*

The participants were reported to Bronx VA medical center at 8am after an overnight fast.  Blood draws was collected in PAXgene™ DNA tubes (PreAnalytiX GmbH) and frozen until DNA extraction.

*Active duty Fort Campbell cohort*

Participants reported to the laboratory after 8:00am at Fort Campbell. 15% of participants were overnight fasting before blood draw. Blood draws were collected in PAXgene™ DNA tubes (PreAnalytiX GmbH) and frozen until DNA extraction.

*MDD UCSF cohort*

Participants were admitted as [outpatients](https://www.sciencedirect.com/topics/medicine-and-dentistry/outpatient) to the UCSF Clinical and Translational Science Institute between 8 a.m. and 11 a.m., having an overnight fasting. Blood draws were collected in PAXgene™ DNA tubes (PreAnalytiX GmbH) and frozen until DNA extraction.

**DNA extraction and methylation array**

Genomic DNA was extracted using the PAXgene Blood DNA Kit (Qiagen, Germantown MD, USA). Genomic DNA (500 ng) was treated with sodium bisulfite using the Zymo EZ96 DNA Methylation Kit (Zymo Research, Orange CA, USA), and genome-wide DNA methylation patterns were profiled using the Infinium HumanMethylation450 BeadChip (450K) Kit (Illumina, Inc., San Diego CA, USA). BeadChips were washed, and allele-specific single-base extension and staining with multiple layers of fluorescence was performed. BeadChip imaging was conducted using the Illumina iScan system (Illumina Inc., San Diego CA, USA). IDAT files containing the raw intensity signals were generated using Illumina’s iControl software.

**Quality control and Normalization**

Four samples from the Discovery cohort were excluded due to low DNA concentrations. All samples passed the log median intensity quality control of methylated and unmethylated channels assessed using the R minfi package v1.30.0^1^. This package assigns a “β value”, to each CpG site that corresponds to the ratio between the fluorescence signal of the methylated allele (C) and the sum of the fluorescent signals of the methylated (C) and unmethylated (T) alleles. As such, a higher β value corresponds to more methylation. Prior to normalization, probes with low detection (average detection p-value > 0.01), located on the X and Y chromosomes, that mapped to multiple locations, and/or that co-located with a SNP, were removed using the ChAMP R package v2.14.0^2^, and resulted in 435,391 DNA methylation probes remained for subsequent analyses.

**Gold Standard BMIQ Normalization**

Illumina 450K methylation chips have type I and II probes, which yield different intensity distributions. The Beta MIxture Quantile normalization (BMIQ) method has been widely applied to adjust the methylation profile to align the distribution of these two probe types within an individual sample^3^. Additionally, quantile normalization is used to adjust between-array difference.

In the supporting material of Horvath (2015)^4^, the author proposed to use an external “gold standard” (e.g. the mean value of a large dataset), instead of an internal quantile normalized standard. This gold standard BMIQ approach is particularly useful in merging data from different resources and platforms, such as Illumina 27K, 450K, and Epic chips. All the arrays from various resources are aligned to the same gold standard, and thereby reduce the batch effect. The new arrays can be processed independently in parallel, without redoing quantile normalization of the whole dataset.

Our gold standard was adapted from the average value of > 3,000 blood samples (mostly young healthy controls) in the Ft Campbell cohort. We compared the robustness against batch effects between the Quantile+BMIQ and the gold standard BMIQ methods. We applied both methods to the Discovery cohort by associating first two principal components of methylation profiles with potential clinical confounders using R swamp package^5^. As shown in Table 1, the higher value standards indicate higher association between principal components to a clinical feature. The white cell percentages (e.g., CD8+ T cell (CD8T), CD4+ T cell (CD4T), Natural Killer(NK), B cell (BC), Monocyte(Mono), and Granulocyte (Gran) Percentage) were estimated by Housemen’s method^6^. The first principal component of gold standard BMIQ normalized data presented a much higher percentage of variance (16.13% vs 8.31%). Moreover, the influence of various white cell compositions was largely reduced by using the gold standard; for example, the impact of granulocyte percentage decreased more than five folds.

**Table 1. Comparison of the robustness against batch effects between the Quantile+BMIQ and gold standard BMIQ approaches**

|  | **Quantile+BMIQ** | | **Gold Standard BMIQ** | | |
| --- | --- | --- | --- | --- | --- |
|  | PC1 (8.31%) | PC2 (5.23%) | PC1 (16.13%) | PC2 (6.46%) |  |
| **PTSD** | 0.001 | 0.000 | 0.007 | 0.000 |  |
| **Site (VA vs NYU)** | 0.032 | 0.001 | 0.012 | 0.021 |  |
| **CD8T %** | 0.218 | 0.150 | 0.024 | 0.363 |  |
| **CD4T %** | 0.246 | 0.000 | 0.001 | 0.085 |  |
| **NK %** | 0.229 | 0.000 | 0.043 | 0.090 |  |
| **BC %** | 0.126 | 0.001 | 0.005 | 0.032 |  |
| **Mono %** | 0.054 | 0.004 | 0.003 | 0.007 |  |
| **Gran %** | 0.701 | 0.028 | 0.129 | 0.364 |  |
| **BMI** | 0.013 | 0.000 | 0.013 | 0.002 |  |
| **Age** | 0.001 | 0.013 | 0.009 | 0.020 |  |

We further generated values from the comparison of 10 technical repeats in the Bronx VA cohort. The gold standard method is also superior in repeatability in terms of the number of probes with large beta value difference.

**Table 2. Repeatability of Quantile+BMIQ and gold standard BMIQ based on 10 technical repeats.**

|  | **Quantile+BMIQ** | **Gold Standard** |
| --- | --- | --- |
| Average number of shifting probes (> 0.2 beta value) | 689 | 293 |
| Average number of shifting probes (> 0.1 beta value) | 7211 | 5856 |

**Quality control**

*Batch effects*

Principal Component Analysis (PCA) analysis was used to identify the dominant variation of genes that were associated with cell composition, site, technical batches, and age (calculated using R-Swamp package^5^). The showed no large batch effect between arrays in Veteran cohorts. The Ft Campbell cohort consisted two large batches, and it was corrected by R limma package v3.40.2^7^.

*Blood cell type proportion prediction:*

Estimated cell types by Housemen’s method^6^ were compared with complete blood counts (CBC), which were processed on the same day of blood collection by CLIA-certified laboratories. Using combination of Discovery and Replication cohort, the estimates and CBC counts were highly correlated. (Lymphocyte *r* = 0.87, Gran *r* = 0.88, Mono *r* = 0.69, NK *r* = 0.68, BC *r* = 0.61, CD4+ T cell *r* = 0.83, and CD8+ T cell *r* = 0.85).

*Smoking genes*

As a measure of quality control, we evaluated the association between the methylation level of three most frequently reported smoking probes^8^ and cotinine level, a metabolite indicator for nicotine exposure. As shown in Fig. 1, all three smoking probes (e.g., cg03636183, cg19859270, cg05575921) were strongly associated with current smoking status in combination of Discovery and Replication cohorts. This suggests current methylation cohorts are of high quality.


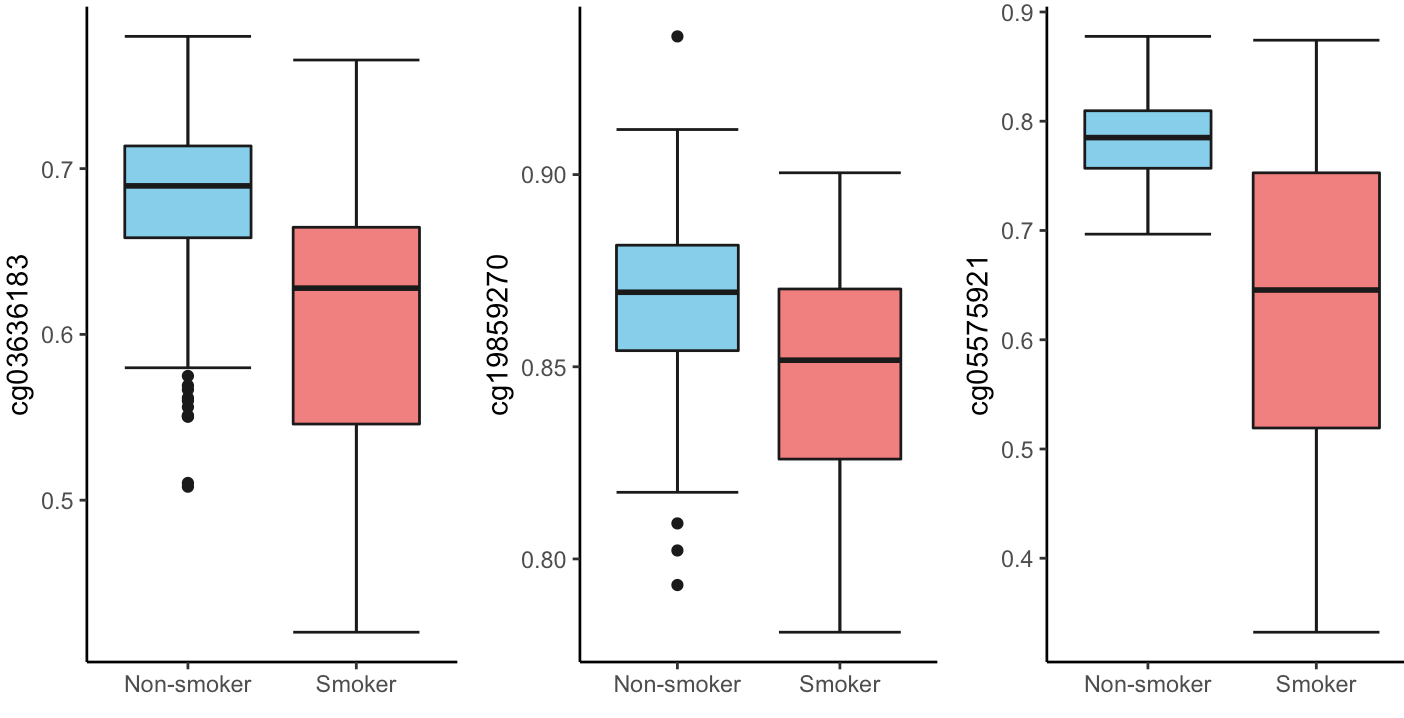


*p* = 3.6e-05

*p* = 2.0e-04

*p* = 1.5e-08

**Figure 1. Smoking genes in veteran cohorts.** Consistent with literature, the non-smokers have significantly greater methylation than smokers for all three smoking genes in combination of Discovery and Replication cohort. The current smoking status of an individual was judged by their cotinine level.

*Race/ethnicity of* PSBC *population*

Infinium PsychArray BeadChip (Illumina Inc., San Diego, CA, USA) was used for genotyping. After a quality-control step, imputation is done with a standard workflow using 1000 genome reference data. PCA was applied to exploratory GWAS genotype data (separately performed on all samples with Illumina SNP arrays) using PLINK^9^ for 275 individuals in the veteran Discovery and Replication cohorts for population race/ethnicity stratification (Figure S6). Based on genetic ancestry, similar ethnicity distributions were found between those with and without PTSD.  Summary statistics from the largest available genome-wide association study (GWAS) of PTSD^10^ (PGC Freeze 2)  (30,000 cases and 170,000 controls) was used. Polygenic risk scoring was conducted with LD (linkage disequilibrium) clumping followed by p-value thresholding procedure. The LD clumping was conducted on windows of 250 kb with allele count squared correlation of 0.1. The P-value thresholding step is done to choose the optimal predictive set of SNPs over a grid of ten equally spaced P-value thresholds from 0.1 to 1. A p-value threshold of 0.5 was found to be optimal. The resulting PRS is a sum of additively coded variants weighted by effect-sizes from the base summary statistics.


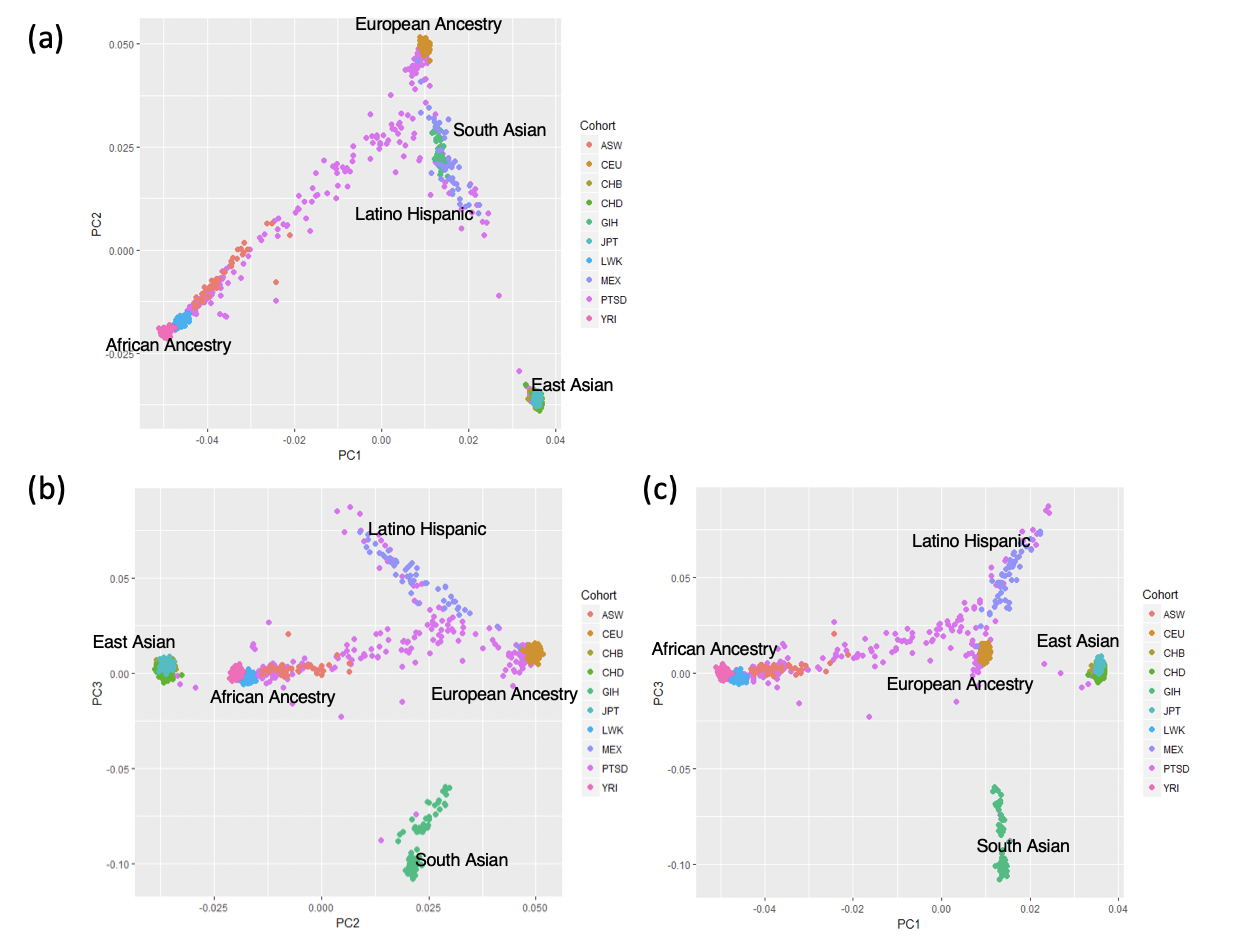


**Figure 2 Principal component analysis (PCA) plots of ethnicity composition of 275** **subjects in the Discovery and Replication cohorts.** Comparison plot of the first three principle components of PTSD cohort and reference ancestry from genome-wide association study (GWAS). Each pink dot represents one individual, and five large colored clusters represents five major ancestral population (African, Latino Hispanic, South Asian, East Asian, and European) The legend denotes the nine ancestral references used in this study (e.g. ASW: African ancestry in Southwest USA; CEU: Utah residents with Northern and Western European ancestry from the CEPH collection; CHB: Han Chinese in Beijing, China; CHD: Chinese in Metropolitan Denver, Colorado; GIH: Gujarati Indians in Houston, Texas; JPT: Japanese in Tokyo, Japan; LWK: Luhya in Webuye, Kenya; MEX: Mexican ancestry in Los Angeles, California; YRI: Yoruba in Ibadan, Nigeria.).

**Stability of the subtyping method**

*Robustness in feature selection*

Dividing 5000 repeats with 10 batches, the top 100 gene regions were very stable (~1% difference between batches), and resulting biotypes were nearly identical. Increasing the number of cross-validation does not improve the results. We also tried different numbers of top gene regions (ranked by the frequency of appearance in 5000 leave-five-out feature selection repeats) ranging from 80 to 120. The supervised classification and biotype assignment on the Discovery and Replication cohorts were then repeated. The assigned biotypes were very stable with > 80% overlap to the original biotypes (100 gene regions) (Table 3) with significant CAPS score difference between two biotypes. Including the intergenic regions will increase more than 30% of candidate features, but it doesn’t improve the predictive power in the training set. For sake of clear biological functions for basis genes and future targeted sequencing panel development, the intergenic regions were filtered.

**Table 3 Similarity of subtypes between different panel sizes and Gene 100.**


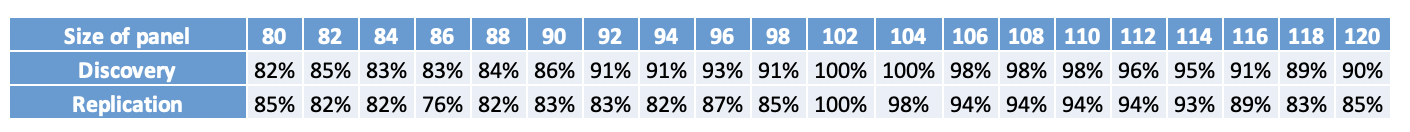


*Robustness in epigenetic estimation of clinical features*

Ranked the 100 gene regions by their absolute weights defined in the equation (1) in *PTSD biotype assignment*, the resulting epigenetic scores were very similar to those based on the 100 gene regions and the biotypes remained identical, using the 70 top-ranked gene regions. Subtypes assigned based on the top 25 gene regions showed approximately 10% deviation from subtypes derived from 100 gene regions. Nevertheless, p-values for differences in CAPS between the two biotypes remained significant in most cases (Table 4).

**Table 4** **Sensitivity analysis on the biotype assignment**


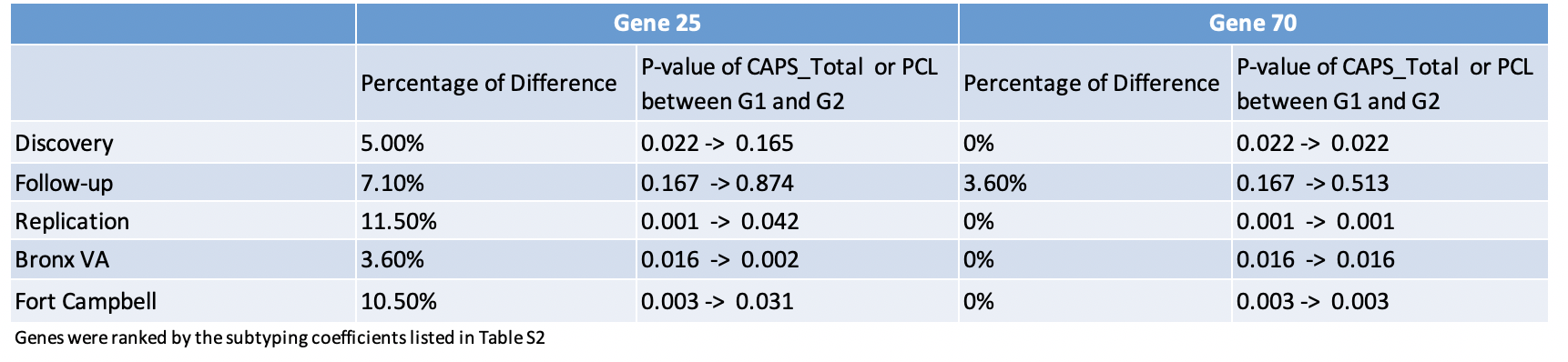


To test the computational robustness of the epigenetic estimators, the leave-one-out (removal of one individual at a time) cross-validation method was applied to reconstruct the epigenetic scores. The average correlation between the resulting 162 DNAm ‘Psychological’ and ‘Physical and Dissociative’ scores were 91% and 80%, respectively. In other words, the DNAm ‘Psychological’ score was very stable, while the DNAm Physical and Dissociative score was slightly less so. However, because ‘Psychological’ scores, as we defined them empirically, have larger weights in the biotype score than ‘Physical and dissociative’ score (1.06 versus 0.15), the resulting biotypes remained stable.

*Supervised clustering vs Unsupervised clustering on subtype assignment*

Two unsupervised clustering methods, hierarchical and k-means, were applied on the two DNAm estimators from the Discovery cohort, followed by training LDA classifiers to construct the separation line between the major clusters^11^. Figure 3 illustrates how the differences from the original biotype assignments were marginal and appear to constitute small shifts of the separation line generated by LDA. However, the unsupervised clusters were not as robust as the direct supervised LDA method. For example, removal of some subjects could result in two clusters primarily separated by positive and negative ‘Physical and dissociative’ scores.


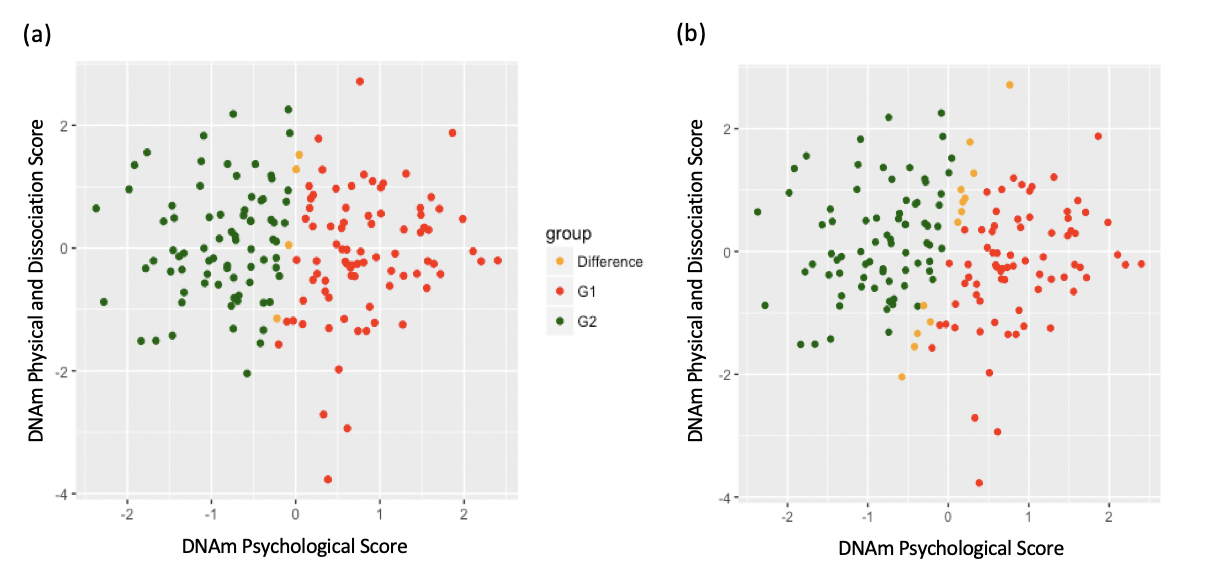


**Figure 3. Comparison of biotypes assigned by unsupervised (a) hierarchical or (b) k-means clustering + LDA methods and direct LDA method.** The resulting scatter plots illustrate 162 individuals in Discovery cohort separated based on their DNAm ‘Psychological’ (x-axis) and ‘Physical and dissociative’ (y-axis) scores. The individuals assigned to G1 by both methods are in green, those assigned to G2 are in red, and those with different assignments are in yellow.

*Technical repeats*

We reprocessed DNA methylation chips of Bronx VA cohort twice with the same extracted DNA and found 75% and 89% of individuals remained the same biotypes. The subtype score was similarly correlated with the current CAPS total scores (Original: N=28, *r* = 0.42, *p* = 0.025, Repeat: N=28, *r* = 0.38, *p* =0.044, Repeat2: N=27, *r* = 0.42, *p* = 0.029).

References

1. Aryee, M.J. et al. Minfi: a flexible and comprehensive Bioconductor package for the analysis of Infinium DNA methylation microarrays. *Bioinformatics* **30**, 1363-1369 (2014).

2. Morris, T.J. et al. ChAMP: 450k chip analysis methylation pipeline. *Bioinformatics* **30**, 428-430 (2013).

3. Teschendorff, A.E. et al. A beta-mixture quantile normalization method for correcting probe design bias in Illumina Infinium 450 k DNA methylation data. *Bioinformatics* **29**, 189-196 (2012).

4. Horvath, S. DNA methylation age of human tissues and cell types. *Genome biology* **14**, 3156 (2013).

5. Lauss, M. et al. Monitoring of technical variation in quantitative high-throughput datasets. *Cancer informatics* **12**, CIN. S12862 (2013).

6. Houseman, E.A., Molitor, J. & Marsit, C.J. Reference-free cell mixture adjustments in analysis of DNA methylation data. *Bioinformatics* **30**, 1431-1439 (2014).

7. Smyth, G.K. in Bioinformatics and computational biology solutions using R and Bioconductor 397-420 (Springer, 2005).

8. Gao, X., Jia, M., Zhang, Y., Breitling, L.P. & Brenner, H. DNA methylation changes of whole blood cells in response to active smoking exposure in adults: a systematic review of DNA methylation studies. *Clinical epigenetics* **7**, 113 (2015).

9. Purcell, S. et al. PLINK: a tool set for whole-genome association and population-based linkage analyses. *Am J Hum Genet* **81**, 559-575 (2007).

10. Nievergelt, C.M. et al. International meta-analysis of PTSD genome-wide association studies identifies sex-and ancestry-specific genetic risk loci. *Nature communications* **10**, 1-16 (2019).

11. Drysdale, A.T. et al. Resting-state connectivity biomarkers define neurophysiological subtypes of depression. *Nature medicine* **23**, 28 (2017).
