# Supplementary material for: Epigenetic biotypes of post-traumatic stress disorder in war-zone exposed veteran and active duty males
Source: Mol Psychiatry. 2020 Dec 18;26(8):4300–14. doi: 10.1038/s41380-020-00966-2 (PMC8550967; doi:10.1038/s41380-020-00966-2)
Supplement: Supplementary file 9 — Additional consortium members [file 41380_2020_966_MOESM9_ESM.docx]

Additional members of The PTSD Systems Biology Consortium include Allison Hoke^1^, Nabarun Chakraborty^1^, Linda Petzold^2^, Gwyneth Wu^3^, Guia Guffanti^4^, Taek-Kyun Kim^5^, Min Young Lee^5^, Linda Bierer^6^

^1^ Medical Readiness Systems Biology, Walter Reed Army Institute for Research, Silver Spring, MD, USA; ^2^Department of Computer Science, University of California, Santa Barbara, CA, USA; ^3^Department of Psychiatry, University of California, San Francisco, USA; ^4^Department of Psychiatry, New York Langone Medical School, NY, USA; ^5^Institute for Systems Biology, Seattle, WA, USA; ^6^Department of Psychiatry, James J. Peters VA Medical Center, Bronx, NY, USA;
